# MOF influences meiotic expansion of H2AX phosphorylation and spermatogenesis in mice

**DOI:** 10.1371/journal.pgen.1007300

**Published:** 2018-05-24

**Authors:** Hanwei Jiang, Qian Gao, Wei Zheng, Shi Yin, Liu Wang, Liangwen Zhong, Asim Ali, Teka Khan, Qiaomei Hao, Hui Fang, Xiaoling Sun, Peng Xu, Tej K. Pandita, Xiaohua Jiang, Qinghua Shi

**Affiliations:** 1 Hefei National Laboratory for Physical Sciences at the Microscale, USTC-SJH Joint Center for Human Reproduction and Genetics, The CAS Key Laboratory of Innate Immunity and Chronic Diseases, School of Life Sciences, CAS Center for Excellence in Molecular Cell Science, University of Science and Technology of China (USTC), Collaborative Innovation Center of Genetics and Development, Hefei, Anhui, China; 2 Department of Radiation Oncology, Houston Methodist Research Institute, Houston, TX, United States; Francis Crick Institute, UNITED KINGDOM

## Abstract

Three waves of H2AX phosphorylation (γH2AX) have been observed in male meiotic prophase I: the first is ATM-dependent and occurs at leptonema, while the second and third are ATR-dependent, occuring at zygonema and pachynema, respectively. The third wave of H2AX phosphorylation marks and silences unsynapsed chromosomes. Little is known about H2AX phosphorylation expands to chromatin-wide regions in spermatocytes. Here, we report that histone acetyltransferase (HAT) MOF is involved in all three waves of H2AX phosphorylation expansion. Germ cell-specific deletion of *Mof* in spermatocytes by *Stra8-Cre* (*Mof* cKO) caused global loss of H4K16ac. In leptotene and zygotene spermatocytes of cKO mice, the γH2AX signals were observed only along the chromosomal axes, and chromatin-wide H2AX phosphorylation was lost. In almost 40% of early-mid pachytene spermatocytes from *Mof* cKO mice, γH2AX and MDC1 were detected along the unsynapsed axes of the sex chromosomes, but failed to expand, which consequently caused meiotic sex chromosome inactivation (MSCI) failure. Furthermore, though RAD51 was proficiently recruited to double-strand break (DSB) sites, defects in DSB repair and crossover formation were observed in *Mof* cKO spermatocytes, indicating that MOF facilitates meiotic DSB repair after RAD51 recruitment. We propose that MOF regulates male meiosis and is involved in the expansion of all three waves of H2AX phosphorylation from the leptotene to pachytene stages, initiated by ATM and ATR, respectively.

## Author summary

Phosphorylation of H2AX is a well-established step in the DNA damage response of somatic cells, and also occurs in germ cells during meiosis. During spermatogenesis, H2AX phosphorylation regulates the formation and repair of programmed DSBs and meiotic silencing of unpaired chromosomes. A critical step in these processes is spreading of H2AX phosphorylation along the chromosomes beyond the initiation site, which occurs by a poorly characterized mechanism. Here, we report that MOF is involved in the proper expansion of H2AX phosphorylation during meiosis. MOF also influences meiotic sex chromosome inactivation by facilitating early pachytene recruitment of MDC1, but is dispensable for BRCA1 or ATR recruitment to axial elements. Furthermore, MOF is involved in proper meiotic recombination and male germ cell development. As well as identifying a new MOF-dependent step for regulating expansion of H2AX phosphorylation, these findings have broad implications for the role of epigenetic modifications in meiosis and human reproductive health.

## Introduction

Meiosis, the process that generates haploid daughter cells, is critical for sexual reproduction in eukaryotes. The key to normal meiotic progression is the fidelity of chromosome interactions, during which homologous chromosomes undergo pairing, synapsis, recombination and proper segregation. The formation and repair of programmed DNA double strand breaks (DSBs) are essential for chromosome dynamics in meiotic prophase I. These DSBs are repaired through two pathways, a synthesis-dependent strand annealing (SDSA) pathway to form non-crossover products and the classical pathway through double Holiday Junction (dHJ) formation to create both crossover and non-crossover products [1,2]. The DSBs that persist until pachynema are more likely to be repaired through the classical pathway to generate crossovers [3]. The mismatch repair complex MLH1-MLH3 is required for the resolution of dHJs to form the class I crossovers that account for approximately 90% of crossovers in mice [4].

The dynamics of γH2AX distribution is one of the major characteristics of meiosis and is commonly used as a marker for meiotic progression. In somatic cells, the phosphorylation of H2AX after DSB formation is first initiated by ATM and/or DNA-PK [5], then expanded by the recruitment of MDC1, which is facilitated by MOF mediated H4K16ac [6–8]. Despite its well-known role in the DNA damage response (DDR), many reports highlight that γH2AX should not be considered simply as a DSB marker [9,10]. In mammalian meiotic prophase I, three waves of H2AX phosphorylation can be observed, all of which are initiated and expanded through pathways that are different from those in somatic cells. During leptonema, γH2AX signals are initiated solely by ATM in a DSB-dependent manner and are distributed throughout the nucleus. This wave of γH2AX signals can be found in wild type (WT) and *Atr* knockout mice [11], but not in mice deficient for *Atm* or *Spo11* [12]. The second wave is initiated by ATR at DSBs. This wave of H2AX phosphorylation can be observed in *Atm-*deficient spermatocytes at early-mid zygonema and is lost when *Spo11* or *Atr* is deleted [11–13]. The third wave is also mediated by ATR and marks chromatin associated with unsynapsed axes at the zygotene/pachytene transition [14,15]. Unlike the first two waves of H2AX phosphorylation expansion at leptonema and zygonema, the third wave can be observed in *Spo11*-deficient spermatocytes [12]. This wave is responsible for meiotic silencing of unsynapsed chromosomes (MSUC), and occurs on the unpaired regions of the sex chromosomes, resulting in meiotic sex chromosome inactivation (MSCI) and formation of the sex body [16]. Mutations causing defective expansion of H2AX phosphorylation in pachytene spermatocytes lead to MSCI failure. This triggers the MSCI checkpoint before late pachynema, which subsequently leads to apoptosis of pachytene spermatocytes [16]. This later wave of H2AX phosphorylation is reported to be initiated by the localization of BRCA1 to the unsynapsed axes [17], which recruits ATR for H2AX phosphorylation and MDC1 for γH2AX signal expansion [11,18]. MDC1 also contributes to the DDR in somatic cells [7,19]. However, spreading of H2AX phosphorylation in leptotene and zygotene spermatocytes is unaltered in *Mdc1*^-/-^ spermatocytes [18], and is therefore regulated by unknown mechanisms.

MOF is a major HAT for histone H4 lysine 16 (H4K16) in somatic cells, and belongs to the MYST family of acetyltransferases [20]. Unlike most histone modifications, acetylation of H4K16 is unique in regulating higher order chromatin structures beyond the level of nucleosomes, and removes the natural barrier to damaged DNA sites to facilitate repair protein access to DNA breaks [21,22]. Consistent with this, MOF is essential for the recruitment of MDC1 [23], a key step in the expansion of H2AX phosphorylation after DSB formation in somatic cells. It has also been reported that MOF is indispensable for the recruitment of BRCA1 and 53BP1 to damage sites after DSB formation [23]. In addition to its known function in recruitment of DDR factors, MOF is essential during DSB repair. In somatic cells, *Mof* knockout results in persistent DSBs and reduces homologous recombination (HR) efficiency, which can be rescued by HDAC inhibition [23,24], demonstrating a crucial role for H4K16ac in HR. H4K16ac is also detected in meiotic cells, and deacetylation of H4K16ac during oocyte maturation is critical for proper chromosome segregation [25]. However, the meiotic function of MOF and H4K16ac in male germ cells has not been reported.

In this study, we generated male mice with germ cell-specific *Mof* deletion mediated by *Stra8-Cre* (*Mof* cKO mice). We show that MOF is involved in the expansion of chromatin-wide H2AX phosphorylation in leptonema and zygonema. MDC1 recruitment and MSCI are also disturbed in *Mof* deleted pachytene spermatocytes, with absence of XY-H2AX phosphorylation expansion, which may partly contribute to the germ cell loss observed in *Mof* cKO mice. RAD51 foci persist in *Mof* cKO spermatocytes until diplonema and are associated with a significant reduction in crossover markers and chiasma formation. Together, these data indicate that the expansion of different waves of H2AX phosphorylation, efficient DSB repair, and the formation of crossovers during male meiosis are affected by MOF, thus highlighting novel roles for this protein.

## Results

### *Mof* is highly expressed in testes particularly in spermatocytes

**Fig 1:**
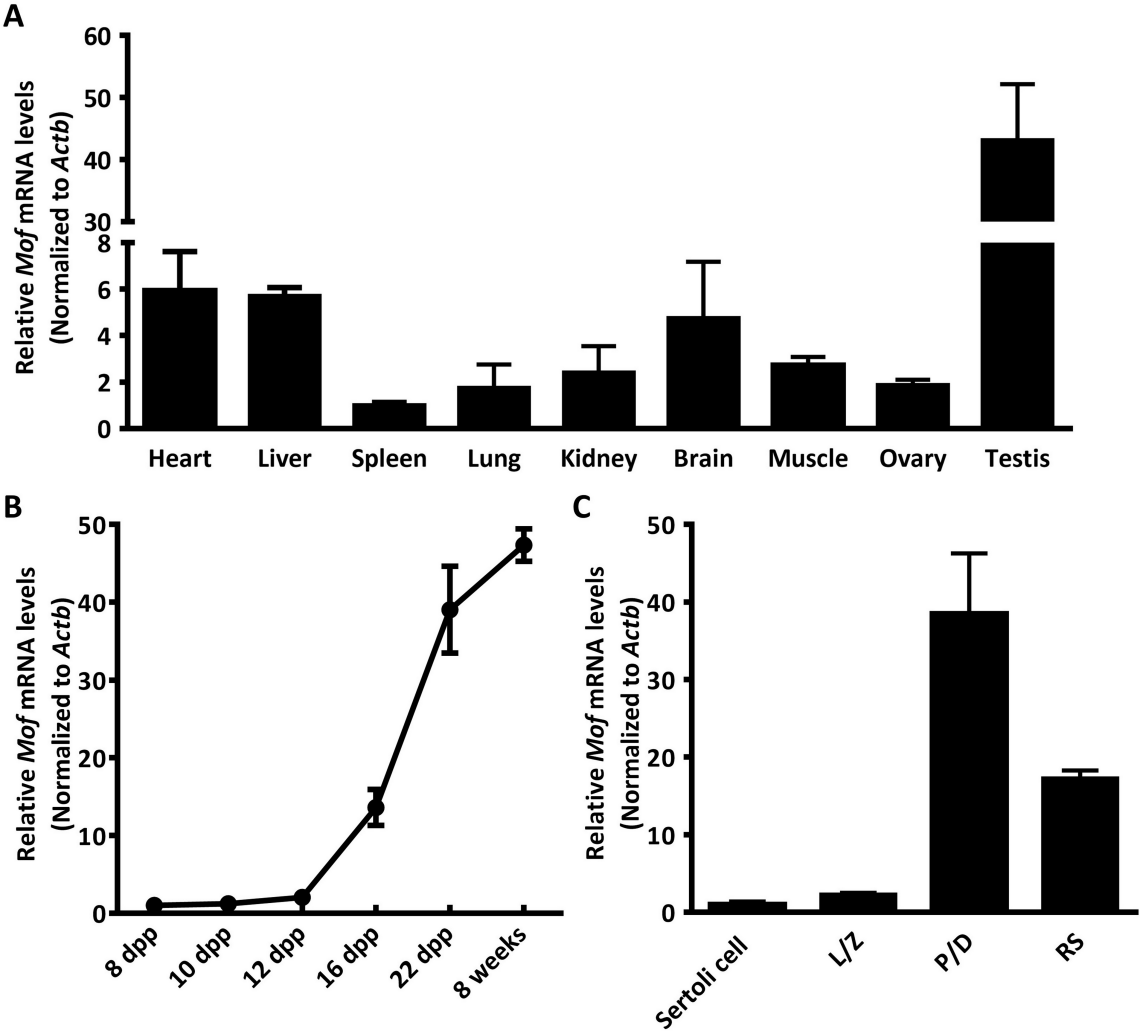
*Mof* is highly expressed in mouse testes and germ cells. A. *Mof* expression in different mouse tissues was detected by Real-time PCR (normalized to *Actb* expressed in the same sample and the value from spleen was set as 1). Data are expressed as mean ± SD from three independent experiments. B. *Mof* expression was detected in testes at indicated ages by Real-time PCR (normalized to *Actb* expressed in the same sample). Data are expressed as mean ± SD from three independent experiments. C. *Mof* expression in isolated spermatocytes, round spermatids and Sertoli cells was detected by Real-time PCR (normalized to *Actb* expressed in the same sample and the value from Sertoli cells was set as 1). L/Z, leptotene/zygotene cell; P/D, pachytene/diplotene cell; RS, round spermatid. Data are expressed as mean ± SD from three independent experiments.

Measurement of mRNA levels by real-time PCR indicated that *Mof* is differentially expressed in mouse tissues, with much higher expression in testes than other organs (Fig 1A). We further analyzed *Mof* expression in testes of mice at different developmental ages. *Mof* mRNA level started to increase at 12 dpp, then dramatically increased from 16 dpp onward, with the highest expression detected in adult testes (Fig 1B), indicating that *Mof* was highly expressed in meiotic and post-meiotic cells. To confirm this, we isolated meiotic prophase I spermatocytes, round spermatids and Sertoli cells from mouse testes (S1 Fig), and analyzed *Mof* expression by real-time PCR. The results showed that *Mof* was highly expressed in pachytene spermatocytes and round spermatids, but had much lower expression in Sertoli cells (Fig 1C).

### *Mof* deletion in spermatocytes reduces testicular size and causes germ cell loss in mice

**Fig 2:**
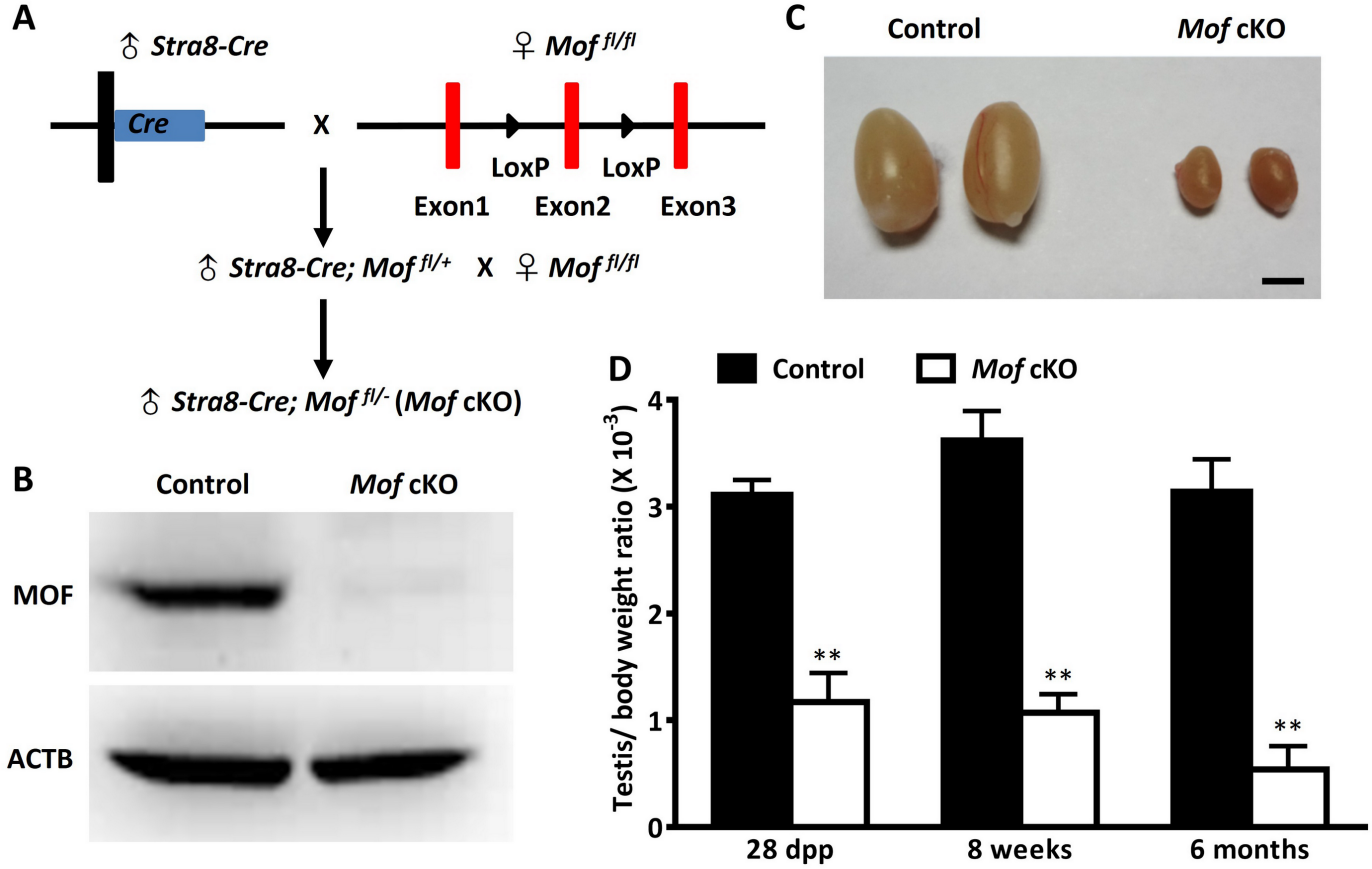
*Mof* specific deletion in germ cells results in reduced testicular size. A. Hybrid scheme used to generate the *Stra8-Cre;Mof* ^*fl/-*^ (*Mof* cKO) mice with *Mof* specifically deleted in germ cells. B. MOF protein level in isolated pachytene/diplotene cells from 8 week old control (*Stra8-Cre*; *Mof* ^*fl/+*^) and *Mof* cKO testes was determined by western blot. ACTB served as a protein loading control. The results shown here are representative data from three independent experiments. C. Representative image of testes from 8 week old control and *Mof* cKO mice. Scale bar, 2 mm. D. Mean ratio of testis/body weight from 28 dpp, 8 week and 6 month old control and *Mof* cKO mice. Three mice were analyzed for each age group, and data are presented as mean ± SD. ** *P*<0.01, Student’s *t*-test.

To determine the functional roles of *Mof* in spermatogenesis, we generated mice with a germ cell-specific *Mof* deletion, by crossing *Mof*-*LoxP* mice (Fig 2A) with *Stra8-Cre* mice, which express CRE recombinase in male germ cells from 3 dpp [26], before they initiate meiosis. Successful deletion of the *Mof* gene was confirmed by the absence of MOF protein in isolated cKO (*Stra8-Cre;Mof* ^*fl/-*^) spermatocytes (Fig 2B) and by loss of H4K16ac in spermatogenic cells (S2 Fig).

**Fig 3:**
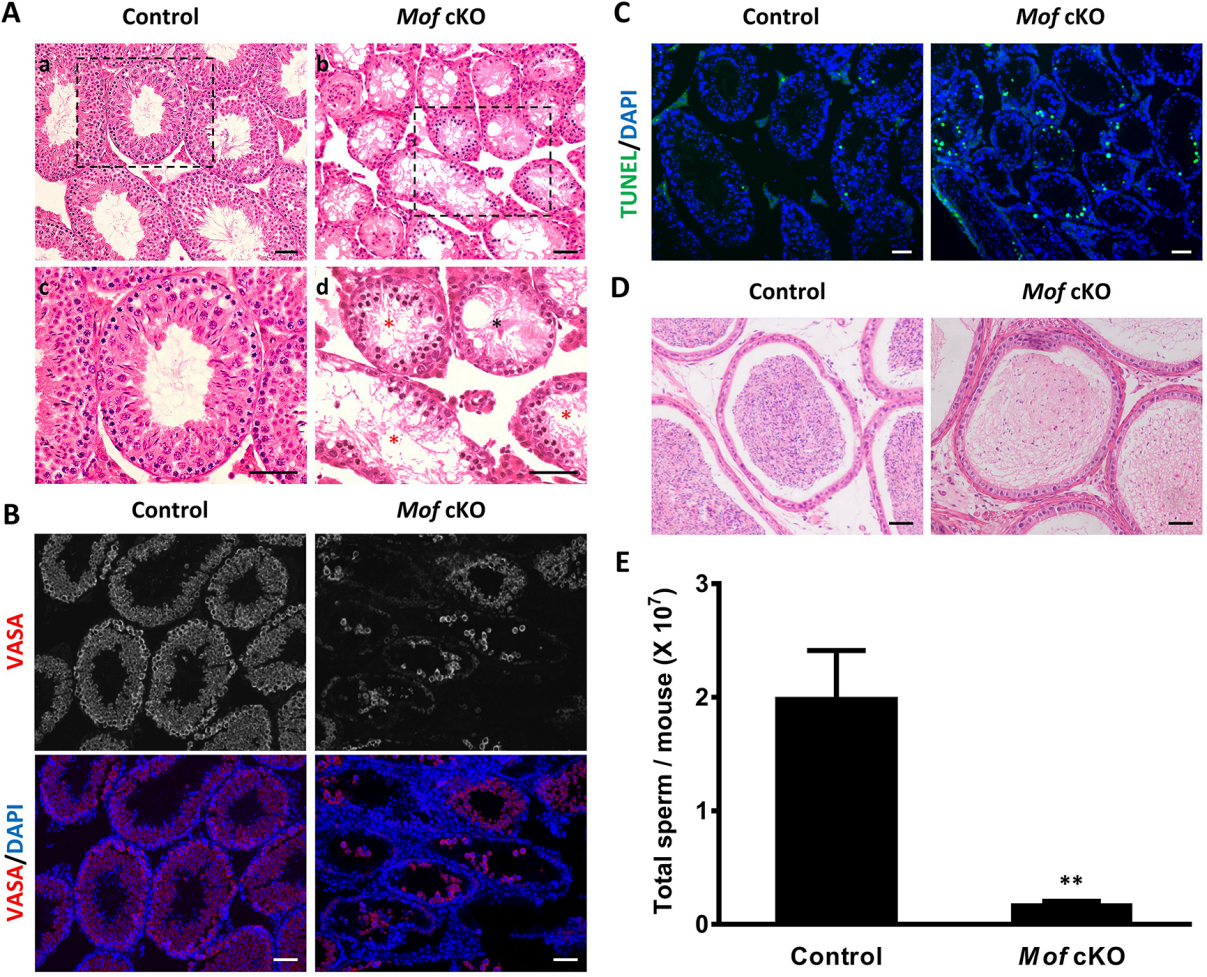
*Mof* cKO mice show massive germ cell loss. A. H&E staining of testes from 8 week old control and *Mof* cKO mice. Normal germ cell arrangement and spermatogenesis are observed in control testes (A, a and c). Massive germ cell loss was observed in *Mof* cKO testes (A, b and d). Black and red stars indicate representative tubules lacking spermatocytes and showing spermatogenic arrest at primary spermatocyte stage, respectively. c and d showed the higher magnification image in rectangular area outlined with black line in a and b. B. Immunofluorescence staining for VASA (a germ cell marker) in control and *Mof* cKO testes of 8 week old mice. C. TUNEL assay of testes from 8 week old control and *Mof* cKO mice. Cells stained green are TUNEL-positive. D. H&E staining of the epididymides from 8 week old control and *Mof* cKO mice. Scale bars, 50 μm. E. Sperm counts in epididymides from 8 week old control and *Mof* cKO mice. Three mice are analyzed for each age group and data are presented as mean ± SD. ** *P*<0.01, Student’s *t*-test.

The *Mof* cKO males had very small testes (Fig 2C), with the ratio of testis to body weight decreased significantly when compared with controls (Fig 2D), suggesting a functional role for *Mof* in spermatogenesis. To further explore this function, we compared testis sections from 8 week old control and *Mof* cKO animals after H&E staining. As shown in Fig 3A, control testes have normal spermatogenesis and elongated spermatids in almost all tubules, while testes from adult *Mof* cKO mice exhibited a marked decrease in the diameter of seminiferous tubules and the number of germ cells, with many tubules showing arrest at the primary spermatocyte stage (Fig 3A, red stars and S3 Fig), and a few tubules lacking meiotic cells (Fig 3A, black stars and S3 Fig). Notably, there were still some tubules with a few round spermatids or even elongated spermatids (S3 Fig), which may partly be due to the incomplete gene deletion in *Mof* cKO mice (S2 Fig). Immunofluorescence (IF) staining of testicular sections for VASA, a germ cell-specific marker [27], indicated massive germ cell loss in *Mof* cKO testes (Fig 3B). Furthermore, TUNEL-positive cells were increased in testes from adult *Mof* cKO mice compared with the control (Fig 3C and S4 Fig). Meiotic progression is also disturbed in *Mof* cKO mice, with a significantly higher ratio of leptotene to zygotene spermatocytes and lower ratio of pachytene spermatocytes compared with the control (S5 Fig), which is consistent with the apoptosis of primary spermatocytes. The sperm count in epididymides of *Mof* cKO mice also dropped to about 10% of that in control animals (Fig 3D and 3E). Altogether, these data demonstrate that *Mof* is involved in the maintenance and development of germ cells at different stages, especially those in meiotic prophase I.

### Chromatin-wide expansion of H2AX phosphorylation fails in *Mof* cKO spermatocytes

**Fig 4:**
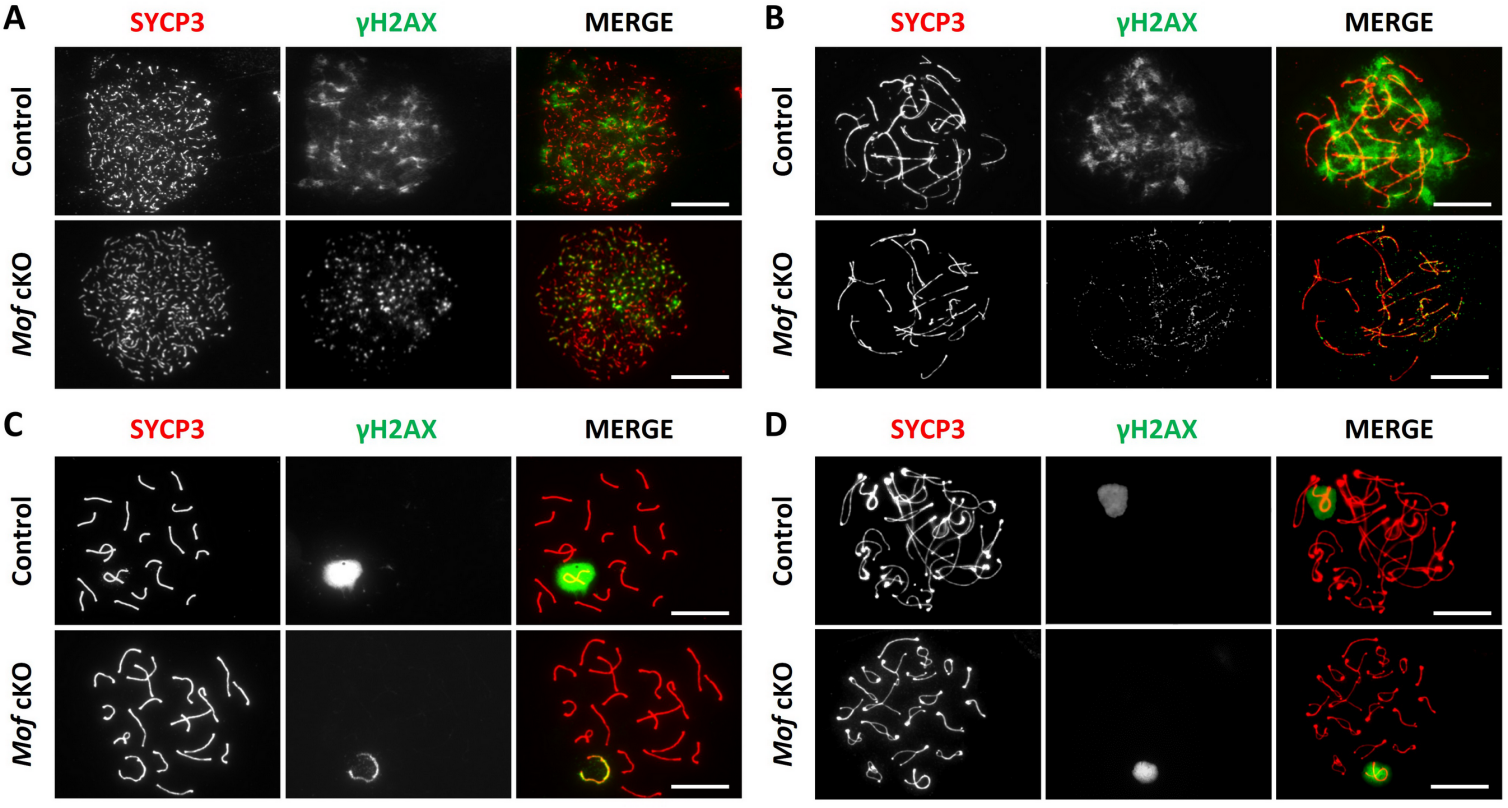
*Mof* deletion disturbs all three waves of chromatin-wide expansion of H2AX phosphorylation during meiosis. Immunofluorescence staining for SYCP3 (red) and γH2AX (green) in control and *Mof* cKO spermatocytes. (A) Leptonema, (B) Zygonema, (C) Pachynema and (D) Diplonema. Scale bars, 10 μm.

The three waves of H2AX phosphorylation expansion are key markers for progression of meiotic prophase I. To further understand how MOF functions in this stage, we analyzed H2AX phosphorylation in control and *Mof* cKO spermatocytes by immunofluorescence staining on surface spread nuclei. All three waves of H2AX phosphorylation in *Mof* cKO spermatocytes were normally initiated, but expansion of the first two expansion waves to the whole chromosomes was blocked in 85.0% of cells at leptonema and 84.5% of cells at zygonema (S6 Fig). Almost no such defective cells were observed in controls (S6 Fig). Instead of forming chromatin-wide signals as in control spermatocytes, the γH2AX signals were observed only on the chromosome axes in leptotene- and along the lateral elements in zygotene-stage *Mof* cKO spermatocytes (Fig 4A and 4B), indicating an important role of MOF in the first two waves of H2AX phosphorylation expansion. Absence of H2AX phosphorylation expansion was observed in 37.2% of *Mof* cKO spermatocytes in early-mid pachytene stage (S6 Fig). The γH2AX signals were confined to the unsynapsed axes and proximal regions of sex chromosomes in these cells, instead of forming sex body as in control mice (Fig 4C). However, this phenotype was not observed in late pachytene and diplotene spermatocytes (Fig 4D and S6 Fig), suggesting that cells with insufficient XY γH2AX are eliminated prior to late pachynema. These results indicate that MOF is involved in all three waves of H2AX phosphorylation expansion during meiosis in male mice.

### *Mof* deletion leads to defects in DSB repair and fewer crossovers in spermatocytes

**Fig 5:**
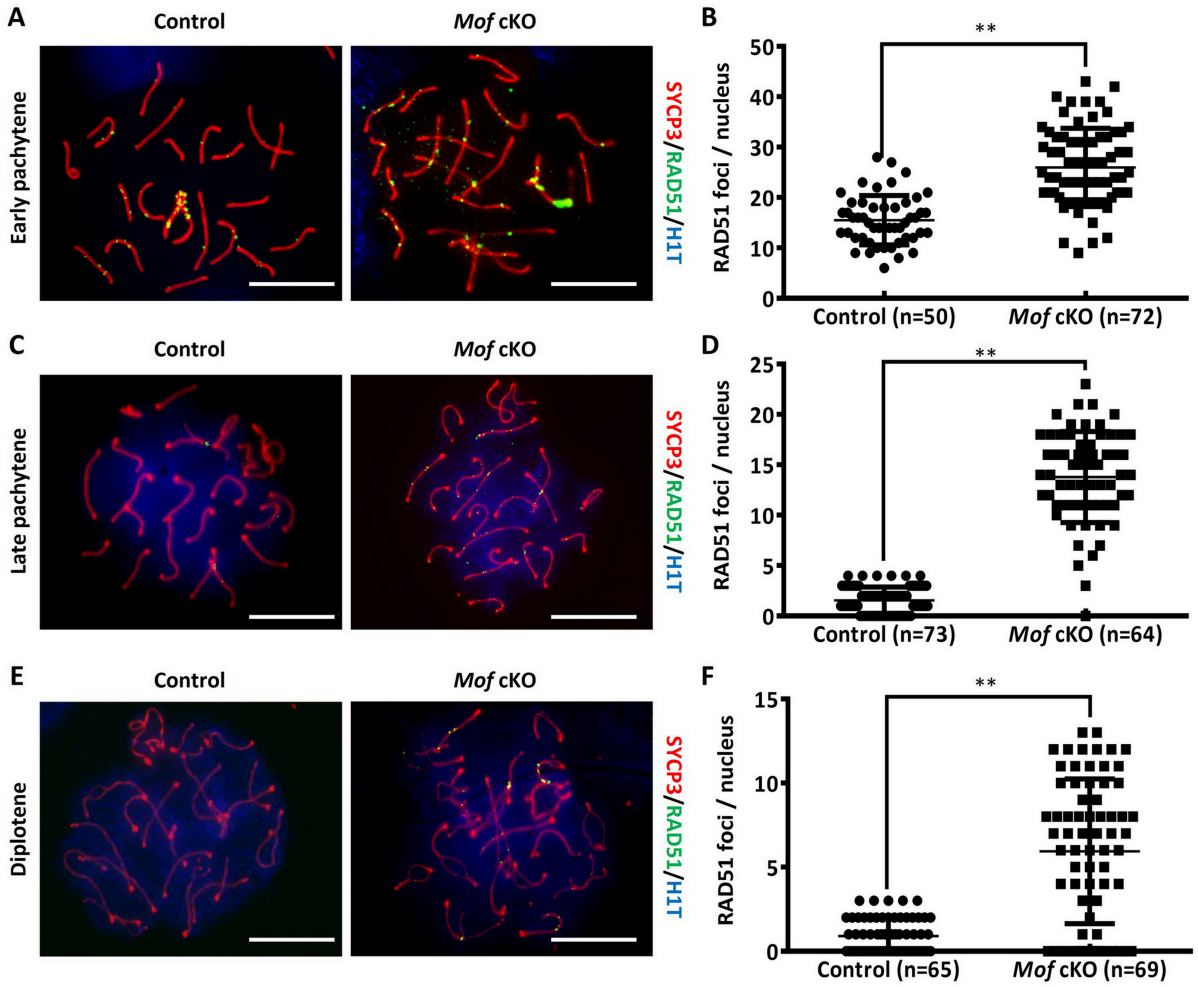
RAD51 foci persist in *Mof* deleted pachytene and diplotene spermatocytes. Immunofluorescence staining for SYCP3 (red), RAD51 (green) and H1T (blue) in control and *Mof* cKO spermatocytes at early pachynema (A), late pachynema (C) and diplonema (E). Scale bars, 10 μm. The mean number of RAD51 foci per cell in control and *Mof* cKO early pachytene (B), mid-late pachytene (D) and diplotene spermatocytes (F). Data are presented as mean ± SD. n, the number of analyzed spermatocytes from 3 mice. ** *P*<0.01, Mann-Whitney test.

One major function for the expansion of H2AX phosphorylation is to recruit proteins related to DSB repair, such as the strand exchange protein RAD51 [28]. Thus, the defect in the first two waves of H2AX phosphorylation expansion in *Mof*-deficient spermatocytes should disturb RAD51 recruitment. To test this, we investigated the accumulation of RAD51 on chromosome axes in *Mof* cKO spermatocytes. Interestingly, in *Mof* cKO leptotene and zygotene spermatocytes, almost the same number of RAD51 foci localized along the meiotic chromosome axes as in control spermatocytes (S7 Fig), indicating that the recruitment of RAD51 was not affected by *Mof* deletion. However, by late pachynema, when most RAD51 foci were lost from paired regions of both autosomes and XY chromosomes in control spermatocytes, *Mof* cKO spermatocytes still exhibited RAD51 foci on axes (Fig 5A and 5C). Some of these foci persisted until diplonema (Fig 5E). Statistical analysis revealed that the number of RAD51 foci in these stages was significantly higher in *Mof* cKO than in control spermatocytes (Fig 5B, 5D and 5F). Similar results were also observed for DMC1 (S8 Fig), indicating that DSB repair in pachynema is compromised in *Mof*-deficient spermatocytes.

**Fig 6:**
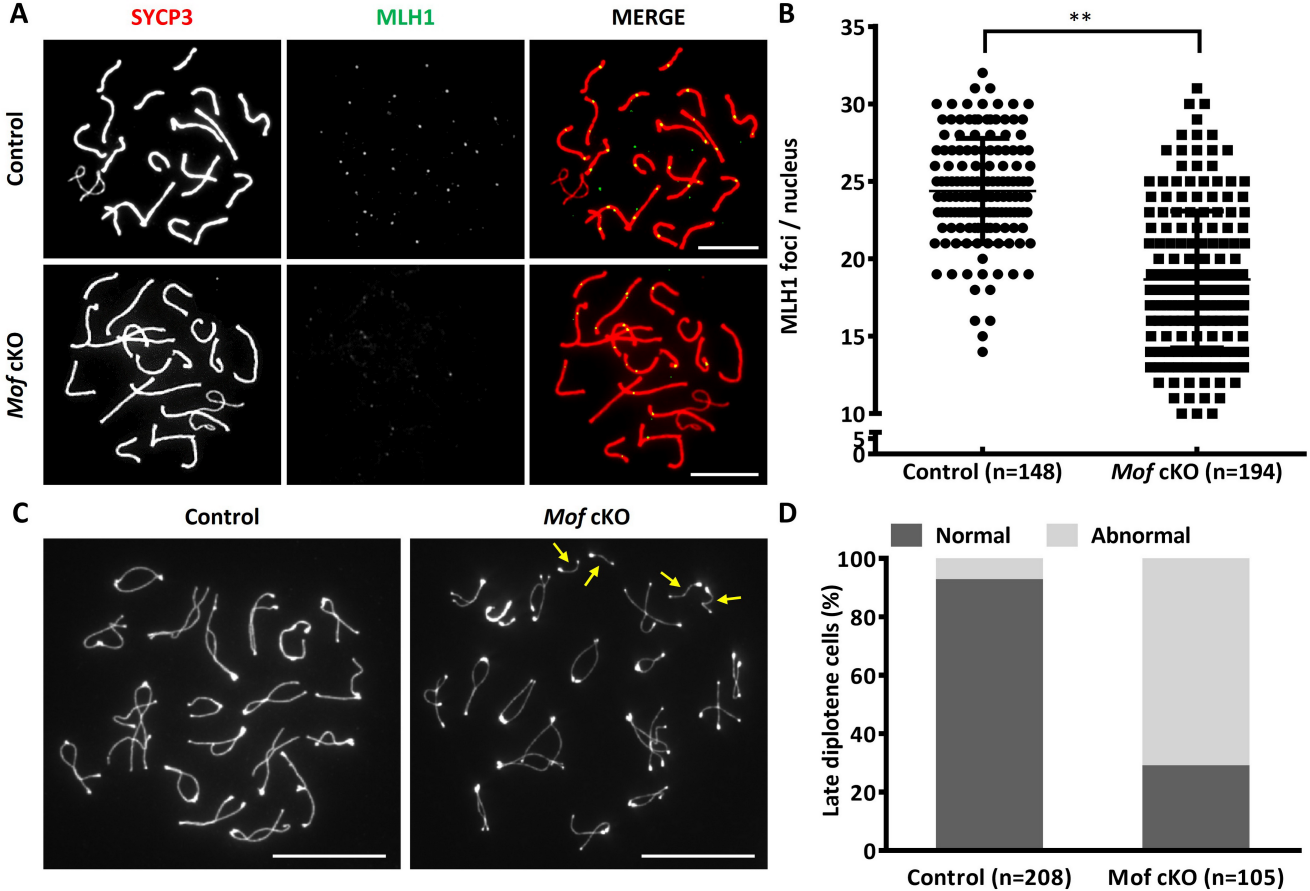
*Mof* cKO mice show reduced homologous recombination. A. Immunofluorescence staining for SYCP3 (red) and MLH1 (green) in control and *Mof* cKO spermatocytes. Scale bars, 10 μm. B. Mean number of MLH1 foci per cell in control and *Mof* cKO spermatocytes. Data are presented as mean ± SD. n, the number of analyzed spermatocytes from 3 mice. ** *P*<0.01, Mann-Whitney test. C. Immunofluorescence staining for SYCP3 in late diplotene spermatocytes (those with at least one pair of completely desynapsed bivalents) from control and *Mof* cKO mice. Arrows show univalent chromosomes with each of them having a typically enlarged telomere at one end and a typically enlarged centromere at another end. Scale bars, 10 μm. D. The ratio of late diplotene cells with (abnormal) or without separated homologous chromosomes (normal) from control and *Mof* cKO mice. n, the number of analyzed spermatocytes from 3 mice.

DSBs repaired in pachytene stage generate at least one crossover on each pair of homologous chromosomes [29]. To determine whether crossover formation was also disturbed by *Mof* deletion, we assessed the number of MLH1 foci in pachytene spermatocytes of *Mof* cKO mice. Consistent with previous reports [30], the average number of MLH1 foci per cell was 24.38 in control pachytene spermatocytes. However, it was reduced to 18.66 in *Mof* cKO spermatocytes (Fig 6A and 6B). As meiosis progresses, crossovers generated in pachytene spermatocytes develop into chiasmata in diplotene spermatocytes [31]. We thus analyzed chiasmata in late diplotene spermatocytes. In *Mof* cKO mice as many as 71.4% of late diplotene spermatocytes bore at least one pair of univalents, which was significantly higher than the 7.7% control level (Fig 6C and 6D). These results collectively show that *Mof* plays an important role in both DSB repair and crossover formation during male meiosis.

### MSCI does not occur in some *Mof* cKO spermatocytes

**Fig 7:**
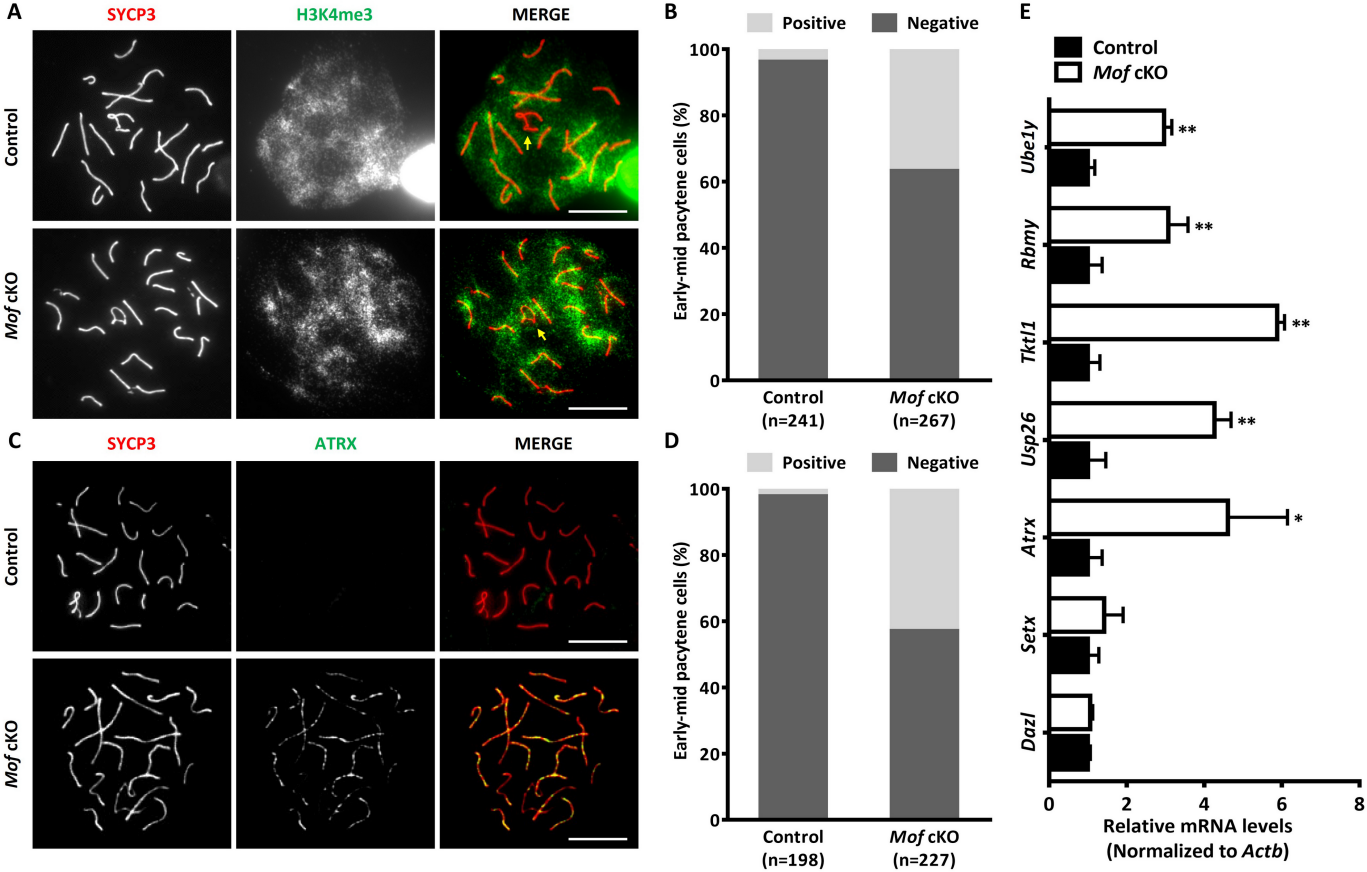
MSCI is disturbed in *Mof* cKO mice. A. Immunofluorescence with SYCP3 (red) and H3K4me3 (green) antibodies in control and *Mof* cKO spermatocytes. Arrows indicate the sex chromosomes. Scale bars, 10 μm. B. The ratio of early-mid pachytene cells with negative (normal) or positive (abnormal) H3K4me3 staining around sex chromosomes from control and *Mof* cKO mice. n, the number of analyzed spermatocytes from 3 mice. C. Immunofluorescence with SYCP3 (red) and ATRX (green) antibodies in control and *Mof* cKO spermatocytes. Scale bars, 10 μm. D. The ratio of early-mid pachytene cells with (abnormal) or without (abnormal) ATRX staining from control and *Mof* cKO mice. n, the number of analyzed spermatocytes from 3 mice. E. Relative levels of X- or Y-linked genes in control and *Mof* cKO pachytene spermatocytes. The gene levels (normalized to *Actb* expressed in the same sample, with values in control spermatocytes designated as 1), were determined by Real-time PCR to assess MSCI. *Dazl* and *Setx* are located on autosomes. *Atrx*, *Usp26* and *Tktl1* are X-linked, and *Rbmy* and *Ube1y* are Y-linked genes. Data are expressed as mean ± SD from three independent experiments. * *P*<0.05, ** *P*<0.01, Student’s *t*-test.

The third wave of H2AX phosphorylation expansion, which happens at pachynema, is a well-known marker for MSCI [32]. To determine whether the failure in chromatin-wide expansion of H2AX phosphorylation in *Mof* cKO spermatocytes would also disturb MSCI, we first examined global transcriptional status in pachytene spermatocytes by immunostaining for trimethylated H3K4 (H3K4me3), an epigenetic modification enriched in transcriptionally activated regions [33]. As reported, the sex chromosomes formed the sex body, from which H3K4me3 was largely excluded, in controls. However exclusion of H3K4me3 was not observed in 36.7% of early-mid pachytene spermatocytes in *Mof* cKO mice (Fig 7A and 7B). To confirm the transcriptional activity of *Mof* cKO sex chromosomes, we measured the distribution of RNA polymerase II (RNA Pol II) in pachytene spermatocytes [34]. Like H3K4me3, RNA Pol II was excluded from the sex body in pachytene spermatocytes in control mice. However, in 38.3% *Mof*-deficient early-mid pachytene spermatocytes, strong RNA Pol II staining was observed around the sex chromosomes (S9 Fig). We also stained pachytene spermatocytes for alpha thalassemia/mental retardation X-linked (ATRX), a protein encoded by an X-linked gene that is silenced by MSCI and not detected in control spermatoyctes. In *Mof* cKO pachytene spermatocytes, ATRX was observed in 42.9% pachytene spermatocytes (Fig 7C and 7D), which further indicated that *Mof* deletion caused MSCI failure.

To further confirm these observations, we analyzed the expression of representative genes on autosomes and unpaired regions of sex chromosomes in isolated pachytene/diplotene cells by real-time PCR. Consistent with the cytological results, a remarkable increase in the expression of X-linked genes such as *Tktl1*, *Usp26* and *Atrx*, was observed in *Mof* cKO spermatocytes when compared with control spermatocytes (Fig 7E). Similar results were found for the Y-linked genes *Ube1y* and *Rbmy* (Fig 7E), whereas expression of genes on autosomes such as *Dazl* and *Setx* remained unaltered by *Mof* deletion (Fig 7E). These results indicate that *Mof* has an important role in MSCI and further corroborate that expansion of H2AX phosphorylation during sex body formation and MSCI in pachytene spermatocytes is facilitated by MOF.

### MOF facilitates the expansion of H2AX phosphorylation by recruiting MDC1 but not BRCA1 in spermatocytes

Facilitation of γH2AX expansion by MOF could occur through two mechanisms: up-regulation of genes related to the expansion or enhanced localization of specific required proteins. Levels of mRNA for genes involved in MSCI including BRCA1, ATR, ATM and MDC1 in isolated spermatocytes did not show significant differences between control and *Mof* cKO spermatocytes (S10 Fig). This indicated that MOF may facilitate sex body formation and MSCI through the recruitment rather than expression of sex body formation related components.

**Fig 8:**
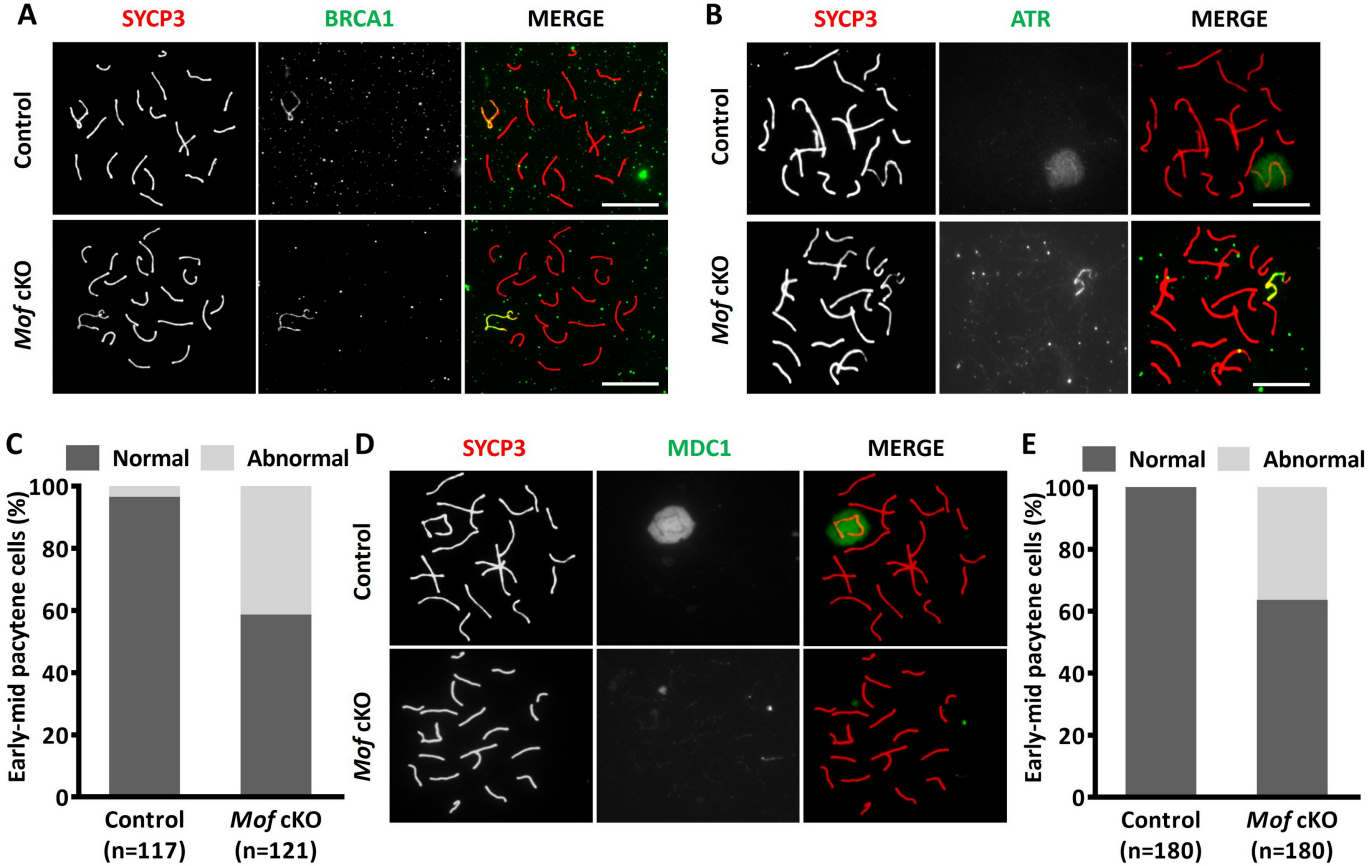
Compromised MDC1 and γH2AX signal expansion in *Mof* deleted pachytene spermatocytes. A. Immunofluorescence with SYCP3 (red) and BRCA1 (green) antibodies in control and *Mof* cKO spermatocytes. Scale bars, 10 μm. B. Immunofluorescence with SYCP3 (red) and ATR (green) antibodies in control and *Mof* cKO spermatocytes. Scale bars, 10 μm. C. The ratio of early-mid pachytene cells with expanded (normal) or liner (abnormal) ATR staining from control and *Mof* cKO mice. n, the number of analyzed spermatocytes from 3 mice. D. Immunofluorescence with SYCP3 (red) and MDC1 (green) antibodies in control and *Mof* cKO spermatocytes. Scale bars, 10 μm. E. The ratio of early-mid pachytene cells with (normal) or without (abnormal) MDC1 staining around sex chromosomes from control and *Mof* cKO mice. n, the number of analyzed spermatocytes from 3 mice.

MSCI begins with recruitment of BRCA1 and ATR along the unpaired axes, followed by MDC1-mediated ATR and H2AX phosphorylation expansion over whole chromatin [18]. To determine whether defective H2AX phosphorylation expansion in *Mof* cKO spermatocytes were caused by the failure in BRCA1, ATR and/or MDC1 recruitment, we first performed immunostaining for these proteins on surface spread spermatocytes from control and *Mof* cKO mice. Surprisingly, BRCA1 was proficiently recruited to the axes of unpaired sex chromosome regions in all *Mof* deleted pachytene cells (Fig 8A), indicating undisturbed MSCI initiation. ATR signals were strong along the unpaired axes and weaker in the sex chromatin regions in almost all control early-mid pachytene spermatocytes (Fig 8B and 8C). In contrast, in more than 40% *Mof* cKO pachytene spermatocytes, ATR staining was confined to the unpaired axes (Fig 8B and 8C). These findings indicate that the defect in sex body formation in *Mof*-deficient spermatocytes is not due to axial recruitment of ATR but to failed expansion of ATR.

We then examined MDC1 localization, to determine whether its recruitment was disturbed by *Mof* deletion. Interestingly, no MDC1 staining was found in 37.1% of *Mof* deleted early-mid pachytene spermatocytes. In control spermatocytes all pachytene cells showed strong MDC1 staining of the sex body (Fig 8D and 8E), indicating that MSCI failure in some *Mof* cKO spermatocytes was due to failed MDC1 recruitment to the sex body.

## Discussion

As a major HAT for H4K16, MOF is well known for its functional role in chromatin remodelling, the DDR and DSB repair. Previous work on HDACs found that H4K16ac deacetylation is critical for proper chromosome segregation and kinetochore function [25]. *Mof* has been found to be indispensable for embryonic stem cell development and its deficiency results in mouse embryonic lethality [35,36]. Our recent study also revealed a role for MOF and H4K16ac in oocyte development [37]. However, *Mof* function in male meiotic cells, especially in the three waves of meiosis-specific H2AX phosphorylation expansion and programmed DSB repair, has not been studied. In this study, we report that *Mof* deletion in spermatocytes using *Stra8-Cre* causes global loss of H4K16ac as well as defective spermatogenesis. In *Mof* cKO mice, the γH2AX signal failed to expand chromatin-wide in leptotene and zygotene spermatocytes, and to the sex body region in about 40% of early-mid pachytene spermatocytes. We also observed persistent RAD51 foci, as well as a significant reduction in crossover number, in *Mof* cKO spermatocytes, consistent with a defect in chiasma formation in *Mof* cKO mice. To be noted, we also observed some tubules with no spermatocytes in cKO testes, which may due to the loss of *Mof* in some spermatogonia, and consequently cause germ cell loss before the initiation of meiosis. Indeed, we did find that *Mof* specific deletion in spermatogonia leads to completely loss of differentiated spermatogonia (S11 Fig).

Our study demonstrates for the first time that one major function of *Mof* in meiotic prophase I is to facilitate the three waves of H2AX phosphorylation expansion. *Mof* deletion blocked the chromatin-wide expansion of H2AX phosphorylation in most leptotene spermatocytes. However, γH2AX foci could still be observed, indicating that *Mof* is not required for the initiation of H2AX phosphorylation at this stage (Fig 4). A similar pattern of γH2AX staining was observed in *Mof* cKO zygotene spermatocytes (Fig 4). We suggest that MOF is involved in the expansion, but not initiation, of the first two waves of H2AX phosphorylation. These two waves of chromatin-wide H2AX phosphorylation expansion are preserved in *Mdc1*-deficient mice [18] and thus MDC1-independent.

The third wave of H2AX phosphorylation expansion is well known for its specific localization and essential role in pachytene spermatocytes. This wave is responsible for MSCI [13]. During the zygotene/pachytene transition, MSCI is initiated by the recruitment of BRCA1 to the axes of Synaptonemal Complexes (SCs) [18,38,39], which consequently recruits ATR and MDC1, leading to phosphorylation and expansion of H2AX [18]. We found that MOF influences the expansion of H2AX phosphorylation during pachynema by recruiting MDC1 to the sex chromosomes. Notably, some *Mof* cKO pachytene spermatocytes exhibit normal γH2AX / MDC1 recruitment, suggesting that *Mof* deletion does not occur in all germ cells. We suspect that some cells that complete pachynema are those with residual MOF. This possibility is supported by the finding that a comparably higher rate of positive H4K16ac staining in surviving Mof cKO diplotene cells compared with pachytene cells (S2C and S2D Fig). The rest of the cells that complete pachynema may come from those pachytene cells with MDC1 recruitment and the expansion of H2AX phosphorylation despite the loss of MOF. This possibility is supported by the fact that H3K4me3, RNA Pol II (S12 Fig) and MDC1 (S13 Fig) staining are similar to controls in the surviving *Mof* cKO diplotene cells, most of which are H4K16ac negative (S2D Fig).

Though H2AX phosphorylation has been reported to facilitate early DSB repair, especially the recruitment of RAD51, we found no defects in these processes in *Mof* cKO spermatocytes (S7 Fig). This result might be explained by the observation that formation of γH2AX foci remains undisturbed in leptotene and zygotene spermatocytes after *Mof* deletion. Notably, at pachynema more RAD51 and DMC1 foci were observed in *Mof* cKO spermatocytes than in controls, suggesting a defect in HR after strand invasion. This is consistent with the decreased number of crossovers in *Mof* cKO spermatocytes, indicating that MOF may participate in processing HR intermediates. One possible mechanism could be via migration of dHJs. It has been reported that H3K4me3 mediated by PRDM9 may facilitate the dHJ migration by creating an extended nucleosome-depleted region (NDR), which restricts the localization of crossovers [40,41]. MOF can recognize H3K4me3 by its chromodomain [42,43], raising the possibility that MOF may recognize the H3K4me3 sites modified by PRDM9 [41], and initiate H4K16ac around that region to stabilize the NDR. Despite their importance in expansion of H2AX phosphorylation, *Mof* and H4K16ac are present at comparably low levels at leptonema and zygonema. We suggest that low levels of *Mof* and H4K16ac during these stages is sufficient to ensure accessibility of DDR factors, as observed in somatic cells [23,42,44], and that higher levels are required at pachynema for DSB repair after stand invasion.

## Materials and methods

### Ethics statement

Animal experiments were carried out under authority of the Institutional Animal Care Committee of the University of Science and Technology of China (Licence USTCACUC1301021). All animal studies were conducted in accordance with the “Guideline on Humane Treatment of Laboratory Animals” (MOST, 2006) and procedures approved by the Institutional Animal Care Committee of the University of Science and Technology of China.

### Experimental mice

*Mof* ^*fl/fl*^ mice, homozygous for a floxed allele of *Mof*, and *Stra8-Cre* transgenic mice were described previously [26,35]. They were mated to generate male *Stra8-Cre;Mof* ^*fl/+*^ mice, which are then crossed with *Mof* ^*fl/fl*^ females. The offsprings were genotyped by PCR and the primers were list in S1 Table. Only the males with *Cre* band, flox band and minus band are considered as *Mof* cKO (*Stra8-Cre;Mof* ^*fl/-*^) mice. All mice were kept under controlled photoperiod conditions (lights-on 08:00–20:00) and supplied with food and double distilled H_2_O *ad libitum*.

### Cell isolation

Spermatogenic and Sertoli cells were isolated as we described previously [45,46]. Briefly, after the mice were euthanized by cervical dislocation, testes were de-capsulated under a dissection microscope. The seminiferous tubules were pooled and washed with PBS thrice and incubated with 1 mg/ml collagenase I (C7661; Sigma, MO, USA) and 0.5 mg/ml DNase I (D4527; Sigma) in Dulbecco modified Eagle medium (DMEM) (12800–017; GIBCO, CA, USA) for 15 min at 34°C on a shaker, then washed twice with DMEM/F12 and further digested with 1 mg/ml collagenase I, 0.5 mg/ml DNase I, 1.5 mg/ml hyaluronidase (H3506; Sigma) and 1 mg/ml trypsin (T8003; Sigma) for 15 min at 37°C. After dissociation, cells were filtered through a nylon mesh (70 μm) and washed with DMEM with 0.5% bovine serum albumin (BSA, A1933; Sigma). Cells were then suspended in 25 ml of 0.5% BSA/DMEM and loaded into the velocity sedimentation apparatus (STA-PUT; Proscience, ON, Canada), and separated on a 2–4% BSA gradient in DMEM for separation by sedimentation at unit gravity. Spermatogenic cells of different substages were identified by phase contrast microscopy and confirmed by immunostaining (S1 Fig), then stored at -80°C until use. For the isolation of Sertoli cells, the final dispersed cells were placed into culture dishes in DMEM/F12 containing 10% fetal bovine serum (HyClone, SV30087-02) and after 1 day culture, the cells were treated with a hypotonic solution (20 mM Tris, pH 7.4) for 1 minute to remove germ cells and and confirmed by immunostaining (S1 Fig). Leptotene/zygotene spermatocytes and Sertoli cells were isolated from 12–14 dpp mice. Pachytene/diplotene spermatocytes and round spermatids were isolated from 8 week mice.

### Western blot

Protein samples from the isolated spermatocytes were prepared in lysis buffer (50 mM Tris/HCl pH 7.4, 300 mM NaCl, 5 mM EDTA, 1% Triton X-100) supplemented with protease inhibitors (04693116001, Roche, Basel, Switzerland). Western blot was carried out as described previously [45] and primary antibodies against MOF (1:500; 20140, Promab, CA, USA) and ACTB (1:1000; MAB374, Millipore, MA, USA) were used to detect the protein bands.

### Hematoxylin and eosin (H&E) staining, immunohistochemistry and TUNEL staining

The control (*Stra-Cre;Mof* ^*fl/+*^) and *Mof* cKO mice were euthanized by cervical dislocation and testes were immediately fixed in Bouin's solution for H&E staining or in 4% paraformaldehyde for immunohistochemistry. For immunohistochemistry, tissue sections were incubated overnight at 4°C with the primary antibody of SOX9 (1:200; AB5535, Millipore), VASA (1:100; ab13840, Abcam, Cambridge, UK) and acetyl Histone H4K16 (1:100; 07–329, Millipore). For H4K16ac staining, sections were treated with metal-3,3’-diaminobenzidine (DAB) after introduction of horseradish peroxidase (HRP) with secondary antibody, and then counterstained with hematoxylin. For VASA staining, sections were incubated with secondary antibody AF488 Donkey Anti-Rabbit IgG (H+L) (1:100, A21206, Molecular Probes, OR, USA) and DAPI. TUNEL staining was performed on testicular sections according to the instructions provided with the cell death detection kit (11684795910, Roche). To reduce inter-experiment variations, testes from control and knockout mice were processed simultaneously. Images were captured using a Nikon Eclipse 80i microscope equipped with a digital camera (Nikon DS-Ri1) or a CCD camera (Hamamatsu) and analyzed using the 694 NIS-Element Microscope imaging software (Nikon).

### Sperm counting

The epididymides and vasa deferentia were removed from 8 week old control and cKO mice, incised several times and incubated in 1 ml buffer containing 75 mM NaCl, 24 mM EDTA and 0.4% BSA (Sigma) at 37°C with 5% CO2 for 30 minutes to allow sperm release from the epididymides. Sperm were collected after a nylon-mesh filtration and counted with a haemocytometer.

### Real-time PCR

RNA isolation, RT-PCR and Real-time PCR were performed as previously described [46,47]. All PCR primers used were listed in S2 Table. For Real-time PCR analysis, Cт values of samples were normalized to the corresponding Cт values of *Actb*. Quantification of the fold change in gene expression was determined by the comparative Cт method.

### Meiotic prophase cell spreading and immunofluorescence staining

Meiotic prophase cell spreading and immunofluorescence staining were prepared as we described previously [48,49]. Briefly, seminiferous tubules were incubated in hypotonic extraction buffer (50 mM sucrose, 17 mM sodium citrate, 30 mM Tris (pH 8.2), 2.5 mM DL-dithiothreitol (DTT), 1 mM phenylmethanesulfonyl fluoride (PMSF, pH 8.3) and 5 mM ethylenediaminetetraacetic acid (EDTA)) on ice for 20 minutes, minced in 100 mM sucrose, and spread on slides dipped in 1% PFA with 0.1% Triton X-100. Slides were incubated in a humid chamber for overnight, dried and washed in PBS and water containing Photoflo (Kodak, NY, USA). Following blocking in 10% donkey serum and 3% BSA, immunofluorescence staining was performed by incubating with primary antibodies of phosphorylated H2A histone family, member X (γH2AX; 1:200; 05–636, Millipore), synaptonemal complex protein 3 (SYCP3; 1:100; AB15093 or AB97672, Abcam), breast cancer 1 (BRCA1; 1:100; SC-1553, Santa Cruz, CA, USA), mediator of DNA damage checkpoint 1 (MDC1; 1:100; AHP799, AbD Serotec, Oxford, UK), ataxia telangiectasia and Rad3 related (ATR; 1:100; SC-1887, Santa Cruz), RNA polymerase II (RNA Pol II; 1:100; AB5408, Abcam), RAD51 recombinase (RAD51; 1:100; SC-8349, Santa Cruz), DNA meiotic recombinase 1 (DMC1; 1:100; SC-22768, Santa Cruz), ATRX (1:150; AB97508, Abcam), trimethyl-Histone H3 (Lys4) (H3K4me3; 1:200; 07–473, Millipore), and MutL homolog 1 (MLH1; 1:50; 551092, BD, NJ, USA) overnight at room temperature, then secondary antibodies at 37°C for 1.5 hour in the dark. Slides were then mounted with cover slips in Vectashield medium. Images were captured using a BX61 microscope (Olympus) connected to a CCD camera and analyzed using the Image-Pro Plus software (Media Cybernetic).

### Statistical analysis

All data are presented as mean ± standard deviation (SD). The Mann-Whitney test was applied to compare the number of RAD51, DMC1 and MLH1 foci between control and *Mof* cKO spermatocytes. The student’s *t*-test was applied to compare the testis/body weight, sperm count, TUNEL and mRNA levels between control and *Mof* cKO mice. Cell population differences between control and *Mof* cKO mice were analyzed by the Chi-square test. Statistical significance was set at *P* < 0.05.

## Supporting information

S1 Fig Immunostaining and purity of isolated cells. Immunostaining and purity (%) of cell populations purified from testes of mice. L/Z, leptotene/zygotene cell; P/D, pachytene/diplotene cell; RS, round spermatid. Scale bars, 50 μm.
(TIF)

S2 Fig *Mof* specific deletion in germ cells results in the loss of H4K16 acetylation in spermatocytes. A. H4K16ac staining of testis sections from 8 week old control and *Mof* cKO mice. Scale bars, 50 μm.
B. Immunofluorescence with SYCP3 (red) and H4K16ac (green) antibodies in control and *Mof* cKO spermatocytes. Scale bars, 10 μm.
C and D. The ratio of pachytene (C) and diplotene (D) spermatocytes in B that are postive or negative for H4K16ac from control and *Mof* cKO mice. n, the number of analyzed spermatocytes from 3 mice.
(TIF)

S3 Fig The severity of tubule degeneration in *Mof* cKO testes. A. Representative tubule degeneration in *Mof* cKO testes at the age of 8 weeks. Note that the *Mof* cKO tubules markedly differ with respect to the most advanced germ cell types. Stars indicate representative tubules and arrows show most advanced germ cells in the tubules. *Scale bars*, 50 μm.
B. Percentages of seminiferous tubules in which spermatogonia, spermatocytes and round spermatids and elongated spermatids represent the most advanced germ cell types in control and *Mof* cKO testes at 8 weeks. Data are presented as mean ± SD. n, the number of analyzed tubules from 3 mice. ** *P*<0.01, Chi-square test.
(TIF)

S4 Fig Cell apoptosis elevation in *Mof cKO* testes. A. Ratios of TUNEL-positive tubules to total examined tubules. B. Average number of TUNEL-positive cells in TUNEL-positive tubules. Data is expressed as mean ± SD for 4 mice and 30–80 round tubules that were randomly selected and scored from testes of each mouse. ** *P* < 0.01, Student’s *t*-test.
(TIF)

S5 Fig Meiotic progression is disturbed in *Mof* cKO mice. Population of spermatocytes at different meiotic substages in control and *Mof* cKO mice. Data are presented as mean ± SD. n, the number of analyzed spermatocytes from 3 mice. * *P*<0.05, ** *P*<0.01, Chi-square test.
(TIF)

S6 Fig The ratio of spermatocytes with defect on the expansion of H2AX phosphorylation at different meiotic substages. A. Immunofluorescence staining for SYCP3 (red) and γH2AX (green) in *Mof* cKO spermatocytes. Images are representative of experiments performed on three biological replicates. Scale bars, 10 μm.
B. The ratio of spermatocytes with defective expansion of H2AX phosphorylation (with H2AX phosphorylation restricted to SCs only) at indicated meiotic substages. Data are presented as mean ± SD. n, the number of analyzed spermatocytes from 3 mice.
(TIF)

S7 Fig DSB formation and RAD51 loading were not affected in *Mof* deleted leptotene and zygotene cells. A and C. Immunofluorescence with SYCP3 (red) and RAD51 (green) antibodies in control and *Mof* cKO spermatocytes at leptotene (A) and zygotene (C) stages. Scale bars, 10 μm.
B and D. The mean number of RAD51 foci per cell in control and *Mof* cKO leptotene(B) and zygotene (D) spermatocytes. Data are presented as mean ± SD. n, the number of analyzed spermatocytes from 3 mice.
(TIF)

S8 Fig DMC1 foci persist in *Mof* deleted pachytene and diplotene spermatocytes. Immunofluorescence with SYCP3 (red) and DMC1 (green) antibodies in control and *Mof* cKO spermatocytes at leptotene (A), zygotene (C), early pachytene (E), mid-late pachytene (G) and diplotene (I) stages. Scale bars, 10 μm.
The mean number of DMC1 foci per cell in control and *Mof* cKO leptotene(B), zygotene (D), early pachytene (F), mid-late pachytene (H) and diplotene (J) spermatocytes. Data are presented as mean ± SD. n, the number of analyzed spermatocytes from 3 mice. ** *P*<0.01, Mann-Whitney test.
(TIF)

S9 Fig RNA Pol II staining showing MSCI failure in *Mof* cKO spermatocytes. A. Immunofluorescence with SYCP3 (red) and RNA Pol II (green) antibodies in control and *Mof* cKO spermatocytes. Arrows indicate the sex chromosomes. Scale bars, 10 μm.
B. The ratio of early-mid pachytene cells with negative (normal) or positive (abnormal) RNA Pol II staining around sex chromosomes from control and *Mof* cKO mice. n, the number of analyzed spermatocytes from 3 mice.
(TIF)

S10 Fig The expression of MSCI related genes remain undisturbed in *Mof* cKO pachytene spermatocytes. The expression of *Brca1*, *Atr*, *Atm* and *Mdc1* mRNAs in isolated pachytene/diplotene spermatocytes from control and *Mof* cKO mice was detected by RT-PCR. *Actb* is used for normalization of the template input and the results shown are representative images from three independent experiments.
(TIF)

S11 Fig Defective spermatogenesis and complete loss of meiotic cells in *Vasa-Cre;Mof* ^*fl/-*^ testes. H&E staining of the testes from 8 week old control and *Vasa-Cre;Mof* ^*fl/-*^ mice. Normal germ cell arrangement and spermatogenesis was observed in control testes. Complete loss of meiotic cells was observed in *Vasa-Cre;Mof* ^*fl/-*^ testes. c and d show the higher magnification image in rectangular area outlined with black line in a and b. Scale bars, 50 μm.
(TIF)

S12 Fig Normal MSCI in *Mof* cKO diplotene spermatocytes. A. Immunofluorescence with SYCP3 (red) and H3K4me3 (green) antibodies in control and *Mof* cKO diplotene spermatocytes. Arrows indicate the sex chromosomes, which are positive or negative for H3K4me3 staining. Scale bars, 10 μm.
B. The ratio of diplotene cells with negative (normal) or positive (abnormal) H3K4me3 staining around sex chromosomes from control and *Mof* cKO mice. n, the number of analyzed spermatocytes from 3 mice.
C. Immunofluorescence with SYCP3 (red) and RNA Pol II (green) antibodies in control and *Mof* cKO diplotene spermatocytes. Arrows indicate the sex chromosomes,with arevpositive or negative RNA Pol II staining. Scale bars, 10 μm.
D. The ratio of diplotene cells with negative (normal) or positive (abnormal) RNA Pol II staining around sex chromosomes from control and *Mof* cKO mice. n, the number of analyzed spermatocytes from 3 mice.
(TIF)

S13 Fig MDC1 staining in *Mof* cKO diplotene spermatocytes. A. Immunofluorescence with SYCP3 (red) and MDC1 (green) antibodies in control and *Mof* cKO diplotene spermatocytes. Arrows indicate the sex chromosomes. Scale bars, 10 μm.
B. The ratio of diplotene cells with normal (positive) or abnormal (negative) MDC1 staining around sex chromosomes from control and *Mof* cKO mice. n, the number of analyzed spermatocytes from 3 mice.
(TIF)

S1 Table Primers used for genotyping. (DOC)

S2 Table Primers used for RT or real-time PCR. (DOC)
