# Supplementary figures and images for: MOF influences meiotic expansion of H2AX phosphorylation and spermatogenesis in mice
Source: PLoS Genet. 2018 May 24;14(5):e1007300. doi: 10.1371/journal.pgen.1007300 (PMC6019819; doi:10.1371/journal.pgen.1007300)

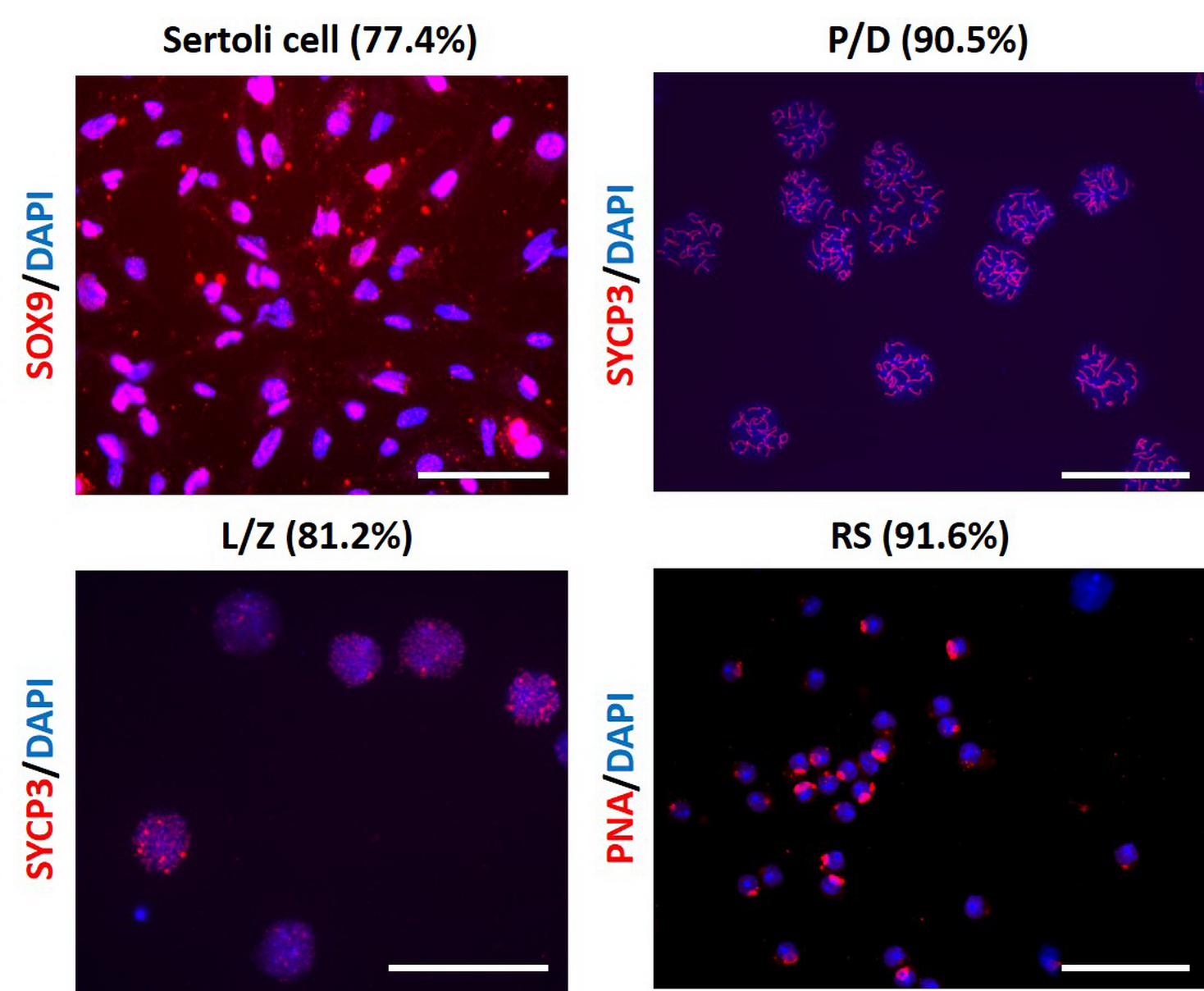

Supplement: S1 Fig — Immunostaining and purity (%) of cell populations purified from testes of mice. L/Z, leptotene/zygotene cell; P/D, pachytene/diplotene cell; RS, round spermatid. Scale bars, 50 μm. (TIF) [file pgen.1007300.s001.tif]

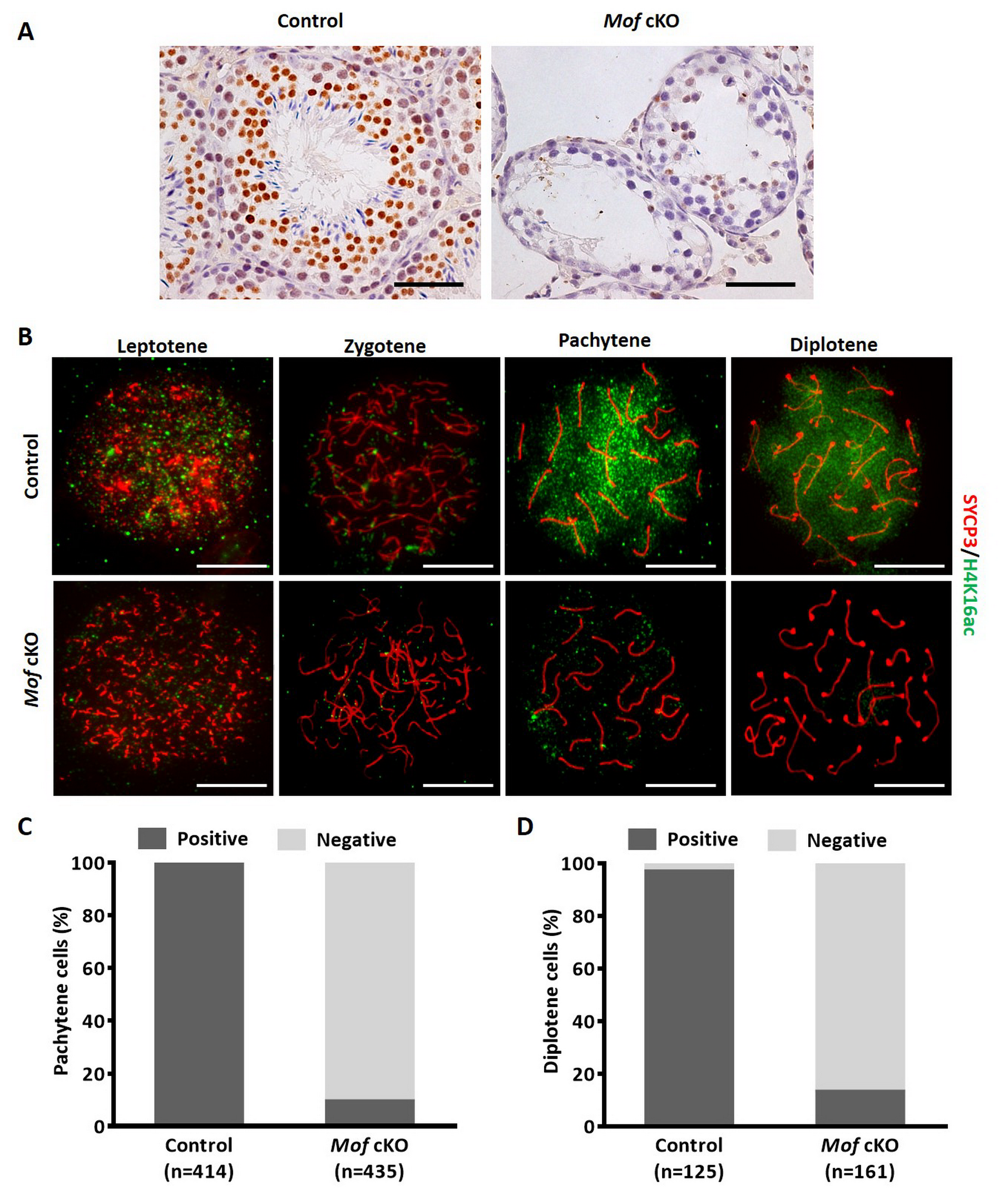

Supplement: S2 Fig — A. H4K16ac staining of testis sections from 8 week old control and Mof cKO mice. Scale bars, 50 μm. B. Immunofluorescence with SYCP3 (red) and H4K16ac (green) antibodies in control and Mof cKO spermatocytes. Scale bars, 10 μm. C and D. The ratio of pachytene (C) and diplotene (D) spermatocytes in B that are postive or negative for H4K16ac from control and Mof cKO mice. n, the number of analyzed spermatocytes from 3 mice. (TIF) [file pgen.1007300.s002.tif]

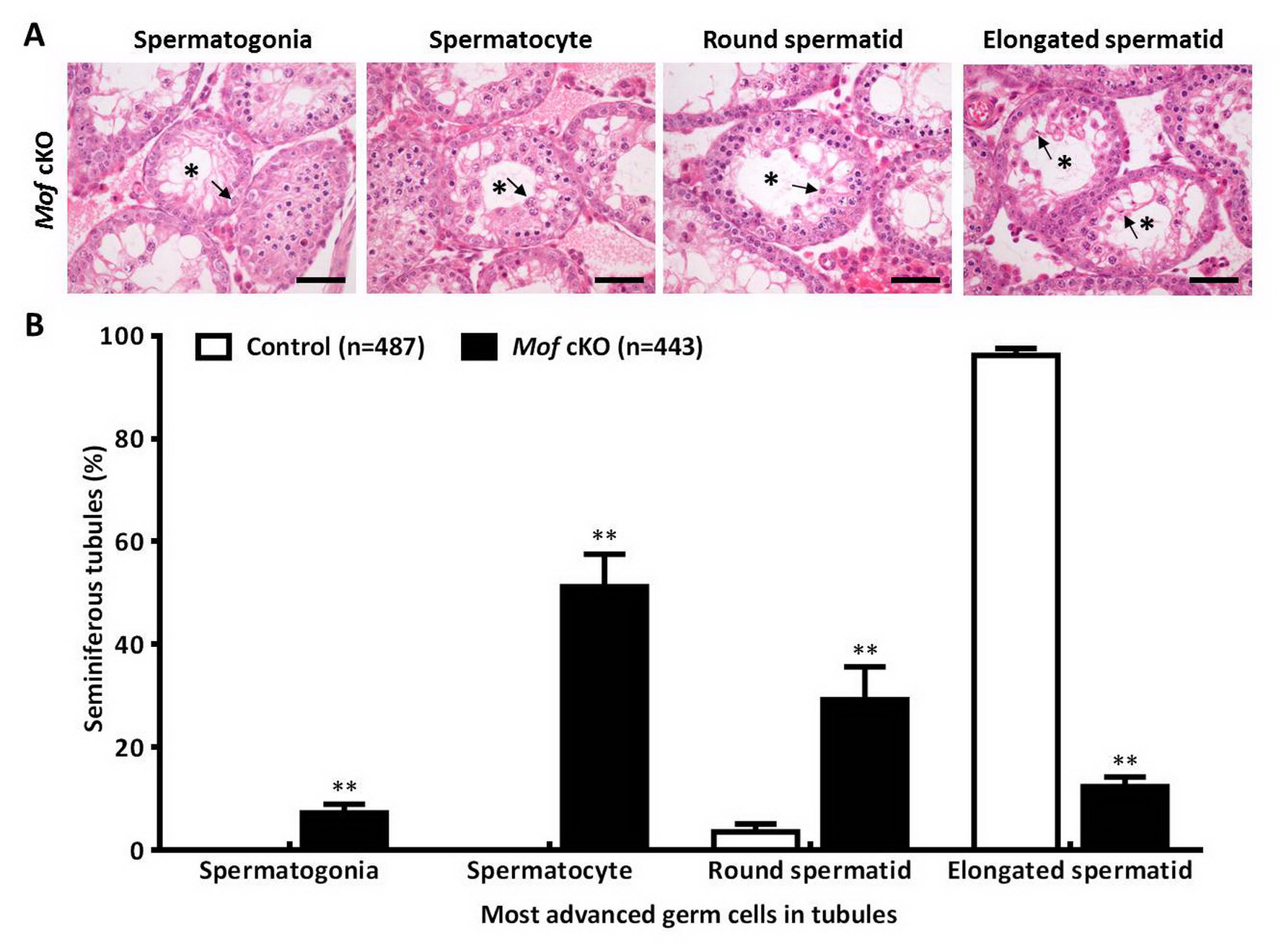

Supplement: S3 Fig — A. Representative tubule degeneration in Mof cKO testes at the age of 8 weeks. Note that the Mof cKO tubules markedly differ with respect to the most advanced germ cell types. Stars indicate representative tubules and arrows show most advanced germ cells in the tubules. Scale bars, 50 μm. B. Percentages of seminiferous tubules in which spermatogonia, spermatocytes and round spermatids and elongated spermatids represent the most advanced germ cell types in control and Mof cKO testes at 8 weeks. Data are presented as mean ± SD. n, the number of analyzed tubules from 3 mice. ** P<0.01, Chi-square test. (TIF) [file pgen.1007300.s003.tif]

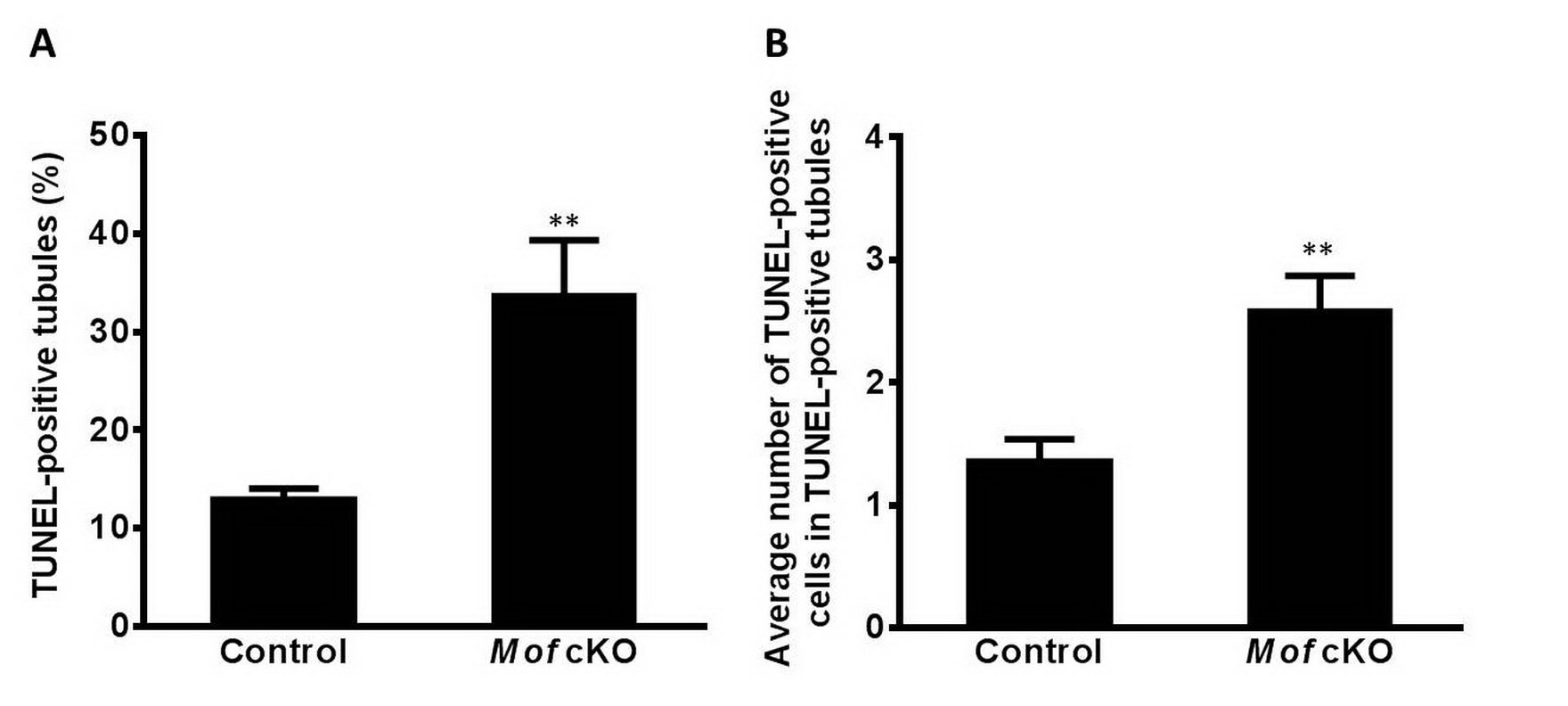

Supplement: S4 Fig — A. Ratios of TUNEL-positive tubules to total examined tubules. B. Average number of TUNEL-positive cells in TUNEL-positive tubules. Data is expressed as mean ± SD for 4 mice and 30–80 round tubules that were randomly selected and scored from testes of each mouse. ** P < 0.01, Student’s t-test. (TIF) [file pgen.1007300.s004.tif]

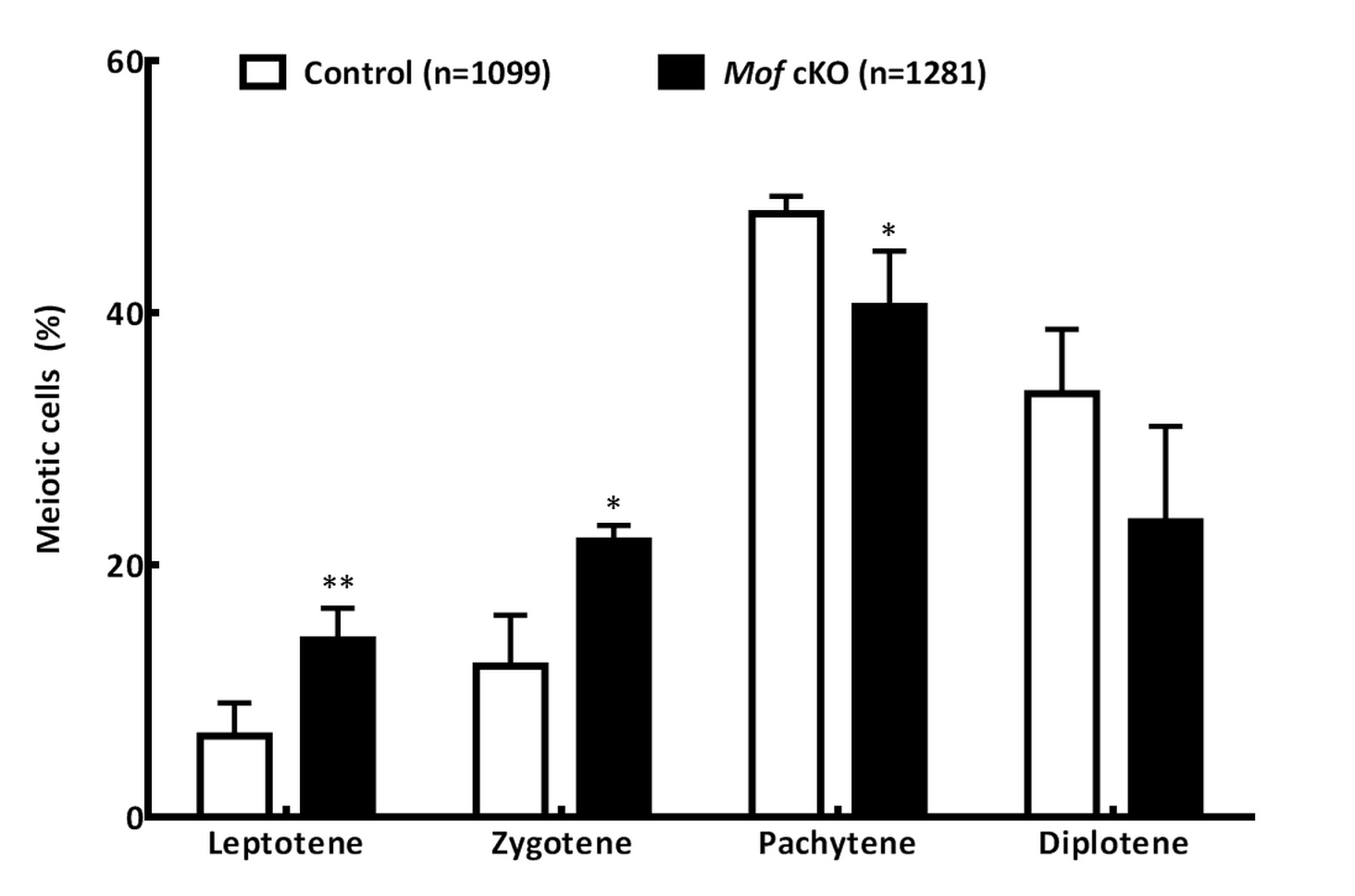

Supplement: S5 Fig — Population of spermatocytes at different meiotic substages in control and Mof cKO mice. Data are presented as mean ± SD. n, the number of analyzed spermatocytes from 3 mice. * P<0.05, ** P<0.01, Chi-square test. (TIF) [file pgen.1007300.s005.tif]

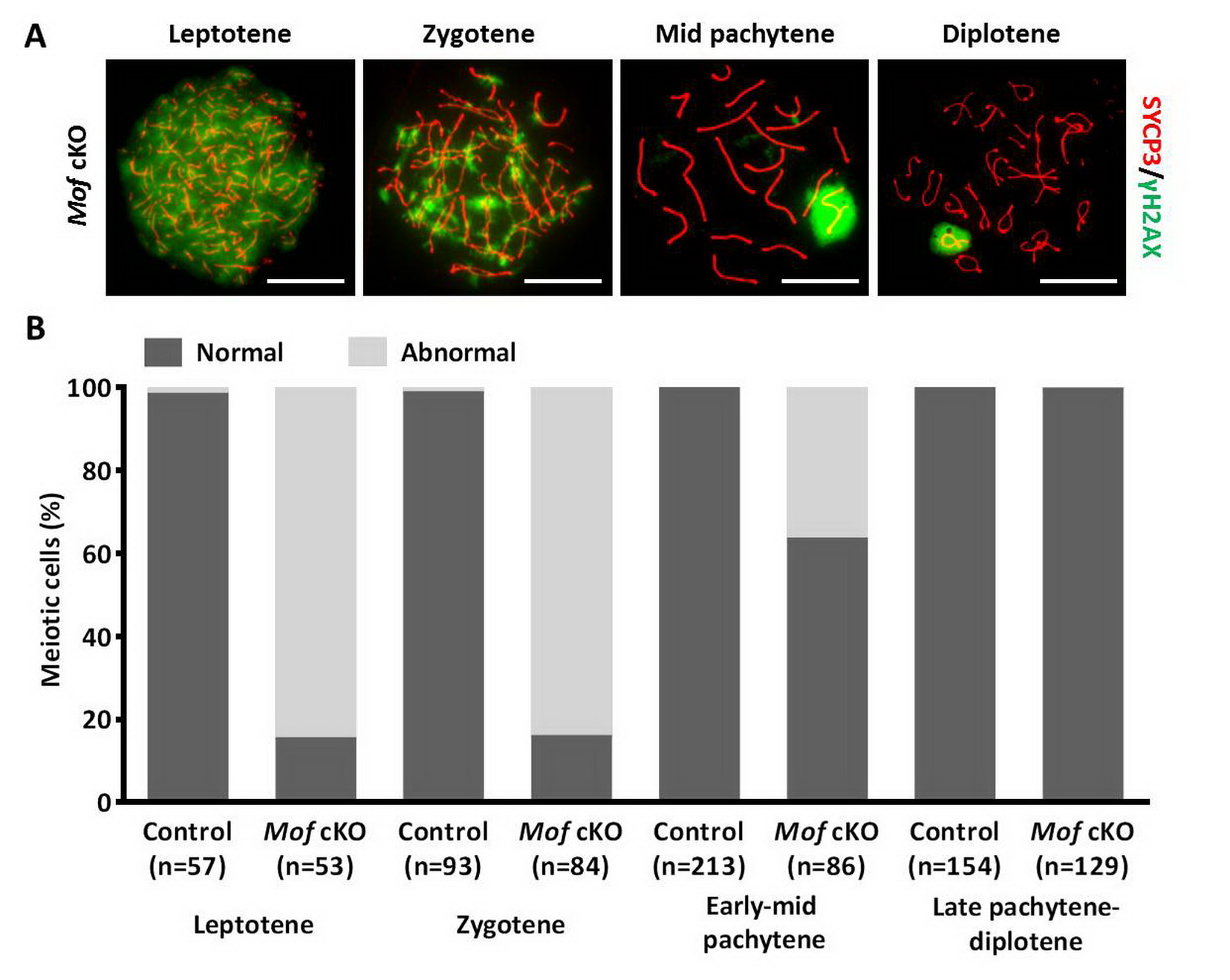

Supplement: S6 Fig — A. Immunofluorescence staining for SYCP3 (red) and γH2AX (green) in Mof cKO spermatocytes. Images are representative of experiments performed on three biological replicates. Scale bars, 10 μm. B. The ratio of spermatocytes with defective expansion of H2AX phosphorylation (with H2AX phosphorylation restricted to SCs only) at indicated meiotic substages. Data are presented as mean ± SD. n, the number of analyzed spermatocytes from 3 mice. (TIF) [file pgen.1007300.s006.tif]

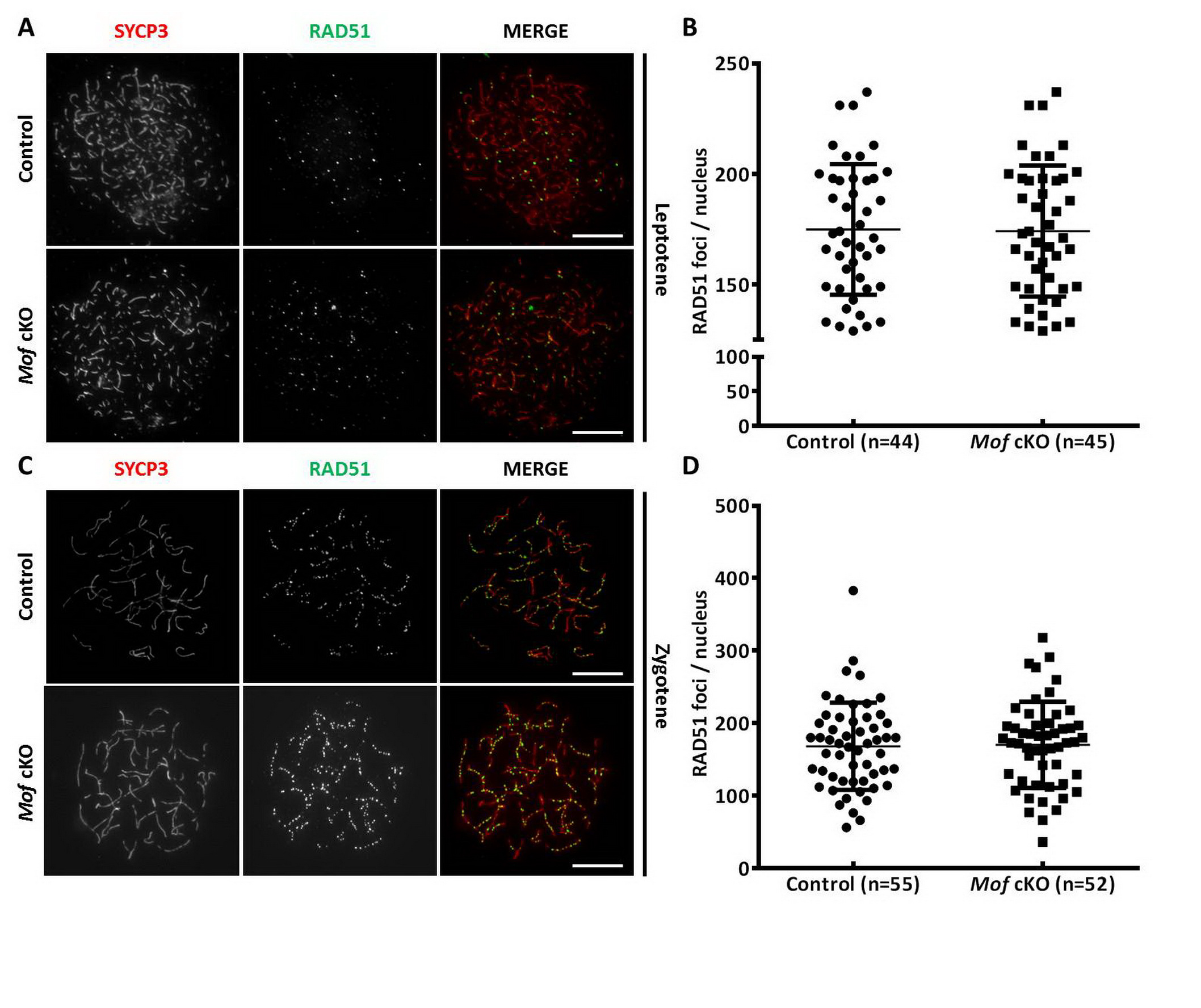

Supplement: S7 Fig — A and C. Immunofluorescence with SYCP3 (red) and RAD51 (green) antibodies in control and Mof cKO spermatocytes at leptotene (A) and zygotene (C) stages. Scale bars, 10 μm. B and D. The mean number of RAD51 foci per cell in control and Mof cKO leptotene(B) and zygotene (D) spermatocytes. Data are presented as mean ± SD. n, the number of analyzed spermatocytes from 3 mice. (TIF) [file pgen.1007300.s007.tif]

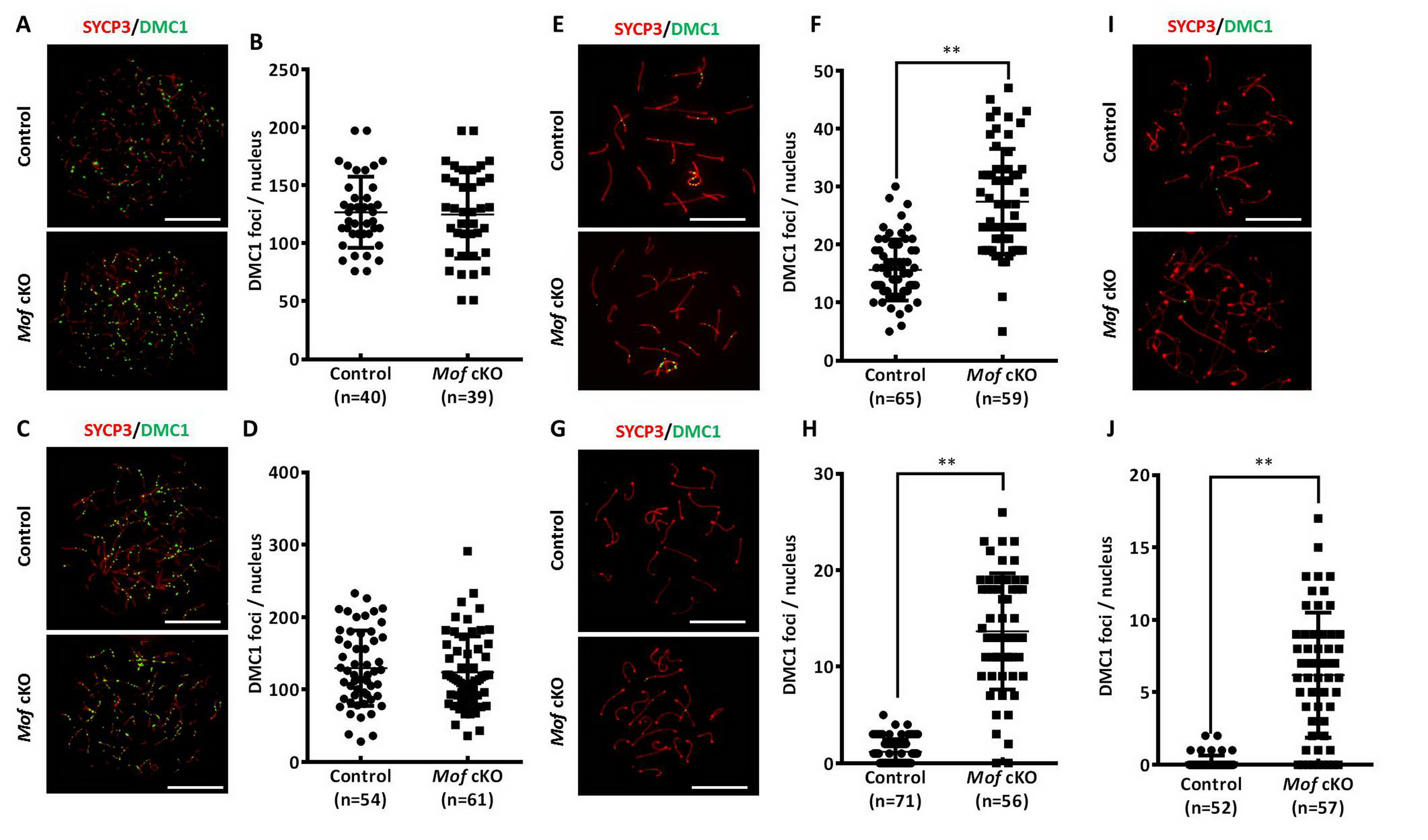

Supplement: S8 Fig — Immunofluorescence with SYCP3 (red) and DMC1 (green) antibodies in control and Mof cKO spermatocytes at leptotene (A), zygotene (C), early pachytene (E), mid-late pachytene (G) and diplotene (I) stages. Scale bars, 10 μm. The mean number of DMC1 foci per cell in control and Mof cKO leptotene(B), zygotene (D), early pachytene (F), mid-late pachytene (H) and diplotene (J) spermatocytes. Data are presented as mean ± SD. n, the number of analyzed spermatocytes from 3 mice. ** P<0.01, Mann-Whitney test. (TIF) [file pgen.1007300.s008.tif]

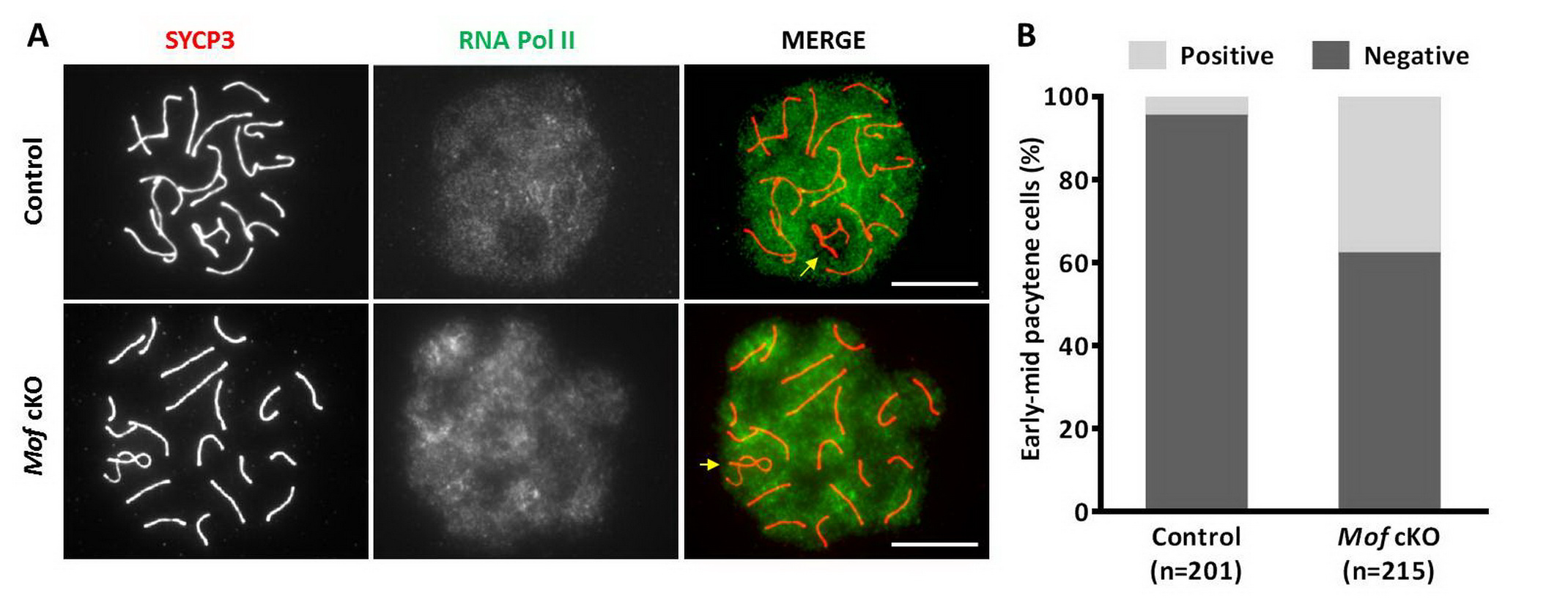

Supplement: S9 Fig — A. Immunofluorescence with SYCP3 (red) and RNA Pol II (green) antibodies in control and Mof cKO spermatocytes. Arrows indicate the sex chromosomes. Scale bars, 10 μm. B. The ratio of early-mid pachytene cells with negative (normal) or positive (abnormal) RNA Pol II staining around sex chromosomes from control and Mof cKO mice. n, the number of analyzed spermatocytes from 3 mice. (TIF) [file pgen.1007300.s009.tif]

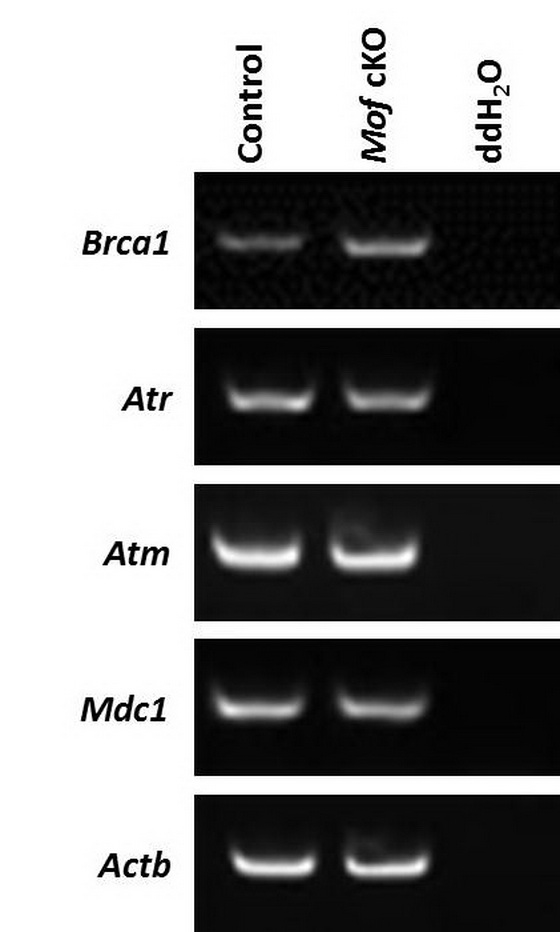

Supplement: S10 Fig — The expression of Brca1, Atr, Atm and Mdc1 mRNAs in isolated pachytene/diplotene spermatocytes from control and Mof cKO mice was detected by RT-PCR. Actb is used for normalization of the template input and the results shown are representative images from three independent experiments. (TIF) [file pgen.1007300.s010.tif]

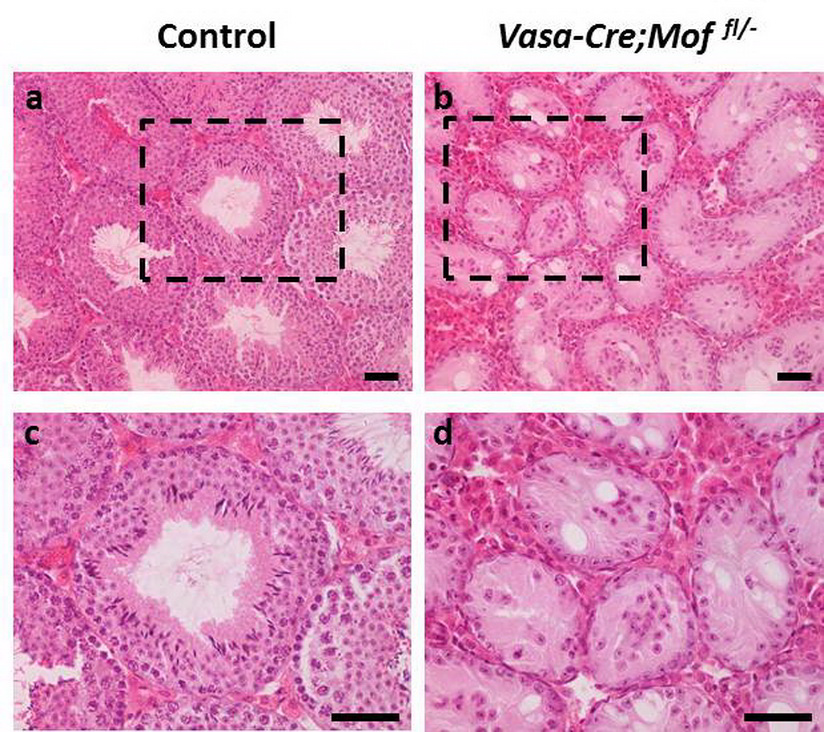

Supplement: S11 Fig — H&E staining of the testes from 8 week old control and Vasa-Cre;Mof fl/- mice. Normal germ cell arrangement and spermatogenesis was observed in control testes. Complete loss of meiotic cells was observed in Vasa-Cre;Mof fl/- testes. c and d show the higher magnification image in rectangular area outlined with black line in a and b. Scale bars, 50 μm. (TIF) [file pgen.1007300.s011.tif]

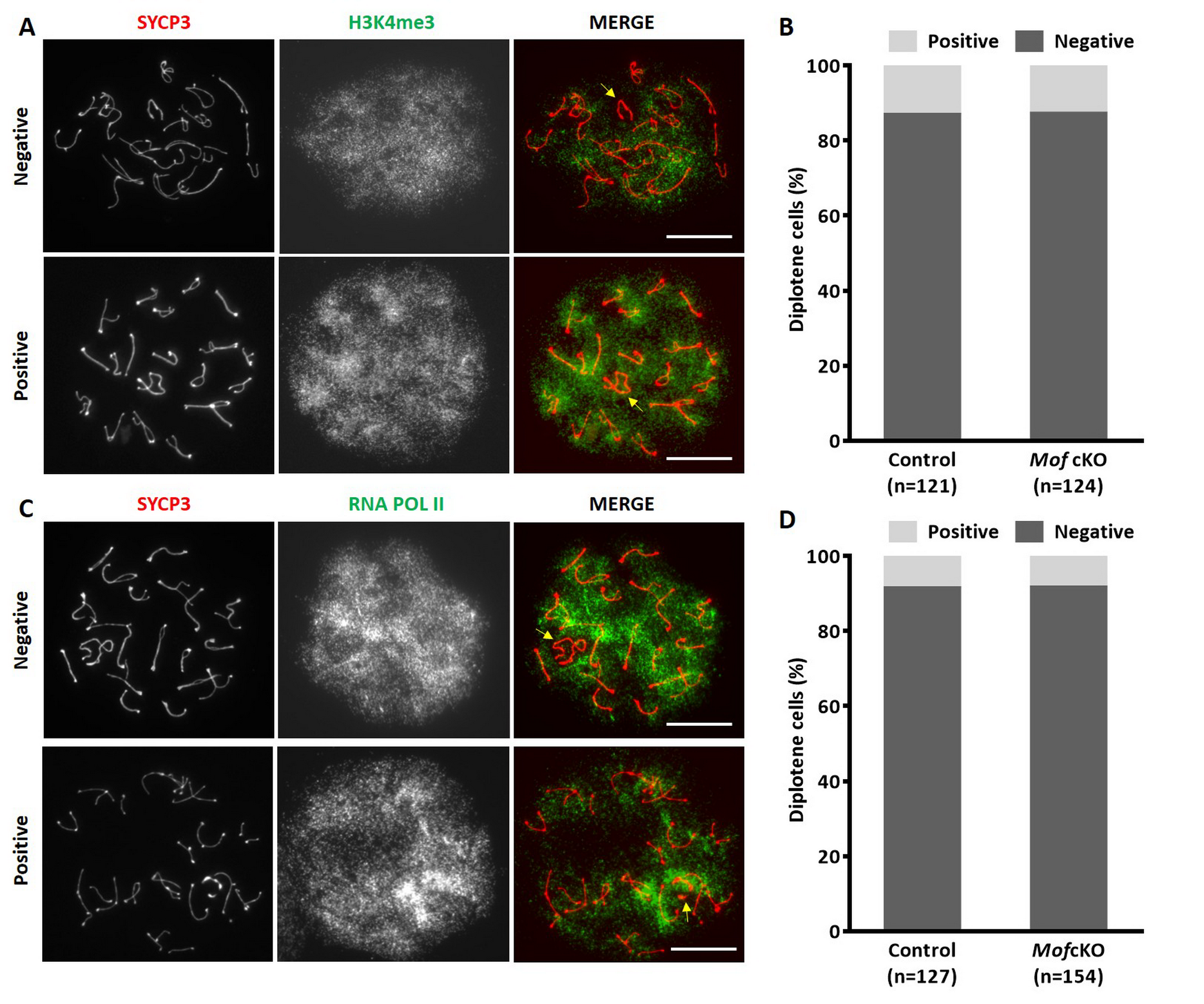

Supplement: S12 Fig — A. Immunofluorescence with SYCP3 (red) and H3K4me3 (green) antibodies in control and Mof cKO diplotene spermatocytes. Arrows indicate the sex chromosomes, which are positive or negative for H3K4me3 staining. Scale bars, 10 μm. B. The ratio of diplotene cells with negative (normal) or positive (abnormal) H3K4me3 staining around sex chromosomes from control and Mof cKO mice. n, the number of analyzed spermatocytes from 3 mice. C. Immunofluorescence with SYCP3 (red) and RNA Pol II (green) antibodies in control and Mof cKO diplotene spermatocytes. Arrows indicate the sex chromosomes,with arevpositive or negative RNA Pol II staining. Scale bars, 10 μm. D. The ratio of diplotene cells with negative (normal) or positive (abnormal) RNA Pol II staining around sex chromosomes from control and Mof cKO mice. n, the number of analyzed spermatocytes from 3 mice. (TIF) [file pgen.1007300.s012.tif]

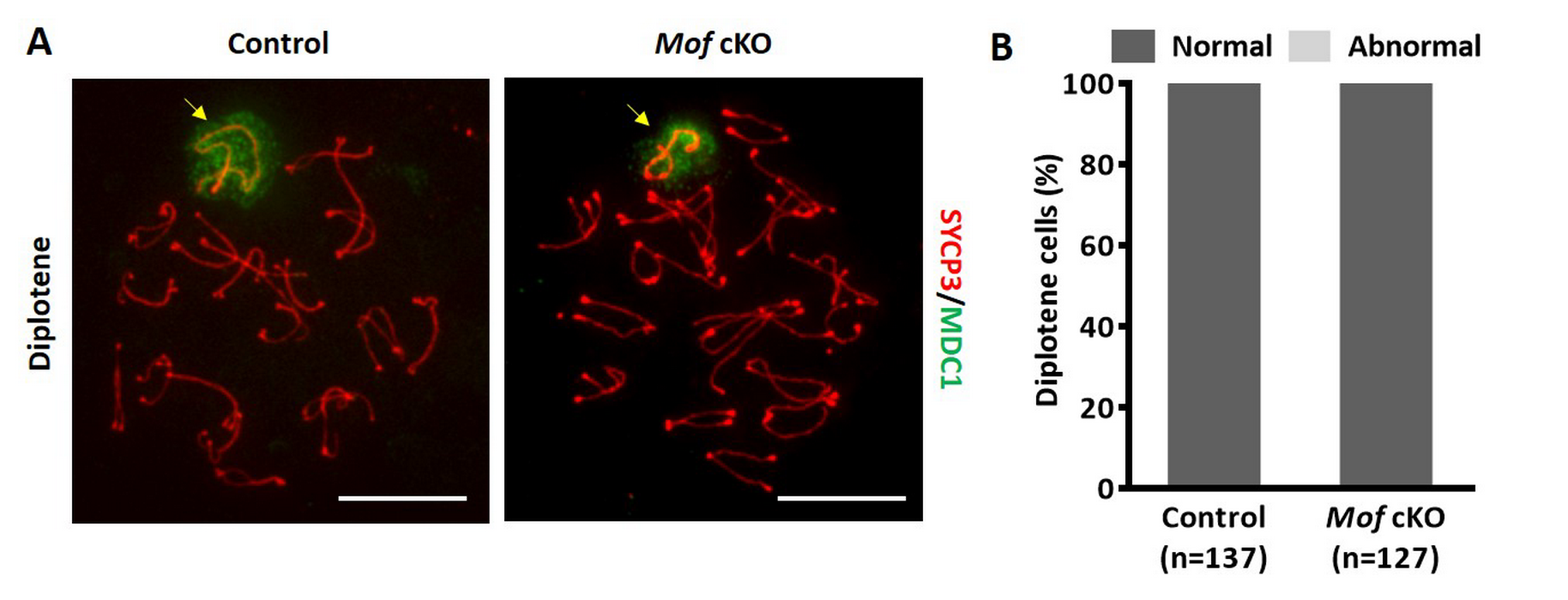

Supplement: S13 Fig — A. Immunofluorescence with SYCP3 (red) and MDC1 (green) antibodies in control and Mof cKO diplotene spermatocytes. Arrows indicate the sex chromosomes. Scale bars, 10 μm. B. The ratio of diplotene cells with normal (positive) or abnormal (negative) MDC1 staining around sex chromosomes from control and Mof cKO mice. n, the number of analyzed spermatocytes from 3 mice. (TIF) [file pgen.1007300.s013.tif]
