# Supplementary material for: MOF influences meiotic expansion of H2AX phosphorylation and spermatogenesis in mice
Source: PLoS Genet. 2018 May 24;14(5):e1007300. doi: 10.1371/journal.pgen.1007300 (PMC6019819; doi:10.1371/journal.pgen.1007300)
Supplement: S1 Table — (DOC) [file pgen.1007300.s014.doc]

**Supplementary Table 1. Primers used for genotyping**

| **Gene name** | **Primers** |
| --- | --- |
| ***Mof*** | **(P1) TGCTGTAGATGGCCTGTGGAGTC** |
|  | **(P2) CCGTTGTGTATCTATGTGAGATG** |
|  | **(P3) ACGGCTGACATGCCTTAGCTTG** |
| ***Stra8-Cre*** | **AGATGCCAGGACATCAGGAACCTG** |
|  | **ATCAGCCACACCAGACACAGAGATC** |
