# Supplementary material for: MOF influences meiotic expansion of H2AX phosphorylation and spermatogenesis in mice
Source: PLoS Genet. 2018 May 24;14(5):e1007300. doi: 10.1371/journal.pgen.1007300 (PMC6019819; doi:10.1371/journal.pgen.1007300)
Supplement: S2 Table — (DOC) [file pgen.1007300.s015.doc]

| **Supplementary Table 2. Primers used for RT or real-time PCR** | | |
| --- | --- | --- |
| **Gene name** | **Forward primers** | **Reverse primers** |
| ***Atr*** | **CCACTGAATGAAACCGGGGA** | **GGGGTCCAACCAAGGTACATC** |
| ***Atm*** | **CCTGACGGTGCCACAGATT** | **CCTTAGGACCTGACTGGGGA** |
| ***Mdc1*** | **AAGACACCCTAACACAAGGGC** | **TCCAAGTCTTTCAACTCCCCAC** |
| ***Brca1*** | **AGGAGAGCCTTCTAATTCCCCA** | **GTATGCCTCAGCAACAGGGA** |
| ***Actb*** | **GCGGACTGTTACTGAGCTGCGT** | **GAAGCAATGCTGTCACCTTCCC** |
| ***Ube1y*** | **ATTGACTTTGAGAAGGATGAC** | **CAGACACACAAGGCCAACTAT** |
| ***Rbmy*** | **AACCGAAGTAACATATACTCA** | **ATCTGCTTTCTCCACGACCTC** |
| ***Usp26*** | **AATGTAACGAAGGGAGAAGTG** | **AGGCTTTGCCTTCTTATCGAG** |
| ***Atrx*** | **GCTTGTGGACAGCAGGTCAAT** | **GTCACGGCTAATATCGTCACTC** |
| ***Dazl*** | **TTCAGGCATATCCTCCTTATC** | **ATGCTTCGGTCCACAGACTTC** |
| ***Setx*** | **AAAATTAGCGCAGAGAAGTCTGG** | **CCAACAGAGCCATCCTTTAATGA** |
| ***Mof*** | **CTGGAAGGGCCAGCATGTTA** | **GGTTAGAGGCCAGGAAACCC** |
| ***Tkt11*** | **TCAAAGGGACTACCATTTGTT** | **AACAGGGGGCGAAGTCATACA** |
|  |  |  |
